# Differential cognitive functioning in the digital clock drawing test in AD-MCI and PD-MCI populations

**DOI:** 10.3389/fnins.2025.1558448

**Published:** 2025-03-13

**Authors:** Chen Wang, Kai Li, Shouqiang Huang, Jiakang Liu, Shuwu Li, Yuting Tu, Bo Wang, Pengpeng Zhang, Yuntian Luo, Tong Chen

**Affiliations:** ^1^School of Medical Technology and Information Engineering, Zhejiang Chinese Medical University, Hangzhou, China; ^2^School of Information Engineering, Hangzhou Medical College, Hangzhou, China; ^3^Zhejiang Engineering Research Center for Brain Cognition and Brain Diseases Digital Medical Instruments, Hangzhou Medical College, Hangzhou, China; ^4^Department of Neurology, The Second Medical Center and National Clinical Research Center for Geriatric Diseases, Chinese PLA General Hospital, Beijing, China

**Keywords:** Alzheimer’s disease, Parkinson’s disease, mild cognitive impairment, digital clock drawing test, cognitive function, digital biomarkers

## Abstract

**Background:**

Mild cognitive impairment (MCI) is common in Alzheimer’s disease (AD) and Parkinson’s disease (PD), but there are differences in pathogenesis and cognitive performance between Mild cognitive impairment due to Alzheimer’s disease (AD-MCI) and Parkinson’s disease with Mild cognitive impairment (PD-MCI) populations. Studies have shown that assessments based on the digital clock drawing test (dCDT) can effectively reflect cognitive deficits. Based on this, we proposed the following research hypothesis: there is a difference in cognitive functioning between AD-MCI and PD-MCI populations in the CDT, and the two populations can be effectively distinguished based on this feature.

**Methods:**

To test this hypothesis, we designed the dCDT to extract digital biomarkers that can characterize and quantify cognitive function differences between AD-MCI and PD-MCI populations. We enrolled a total of 40 AD-MCI patients, 40 PD-MCI patients, 41 PD with normal cognition (PD-NC) patients and 40 normal cognition (NC) controls.

**Results:**

Through a cross-sectional study, we revealed a difference in cognitive function between AD-MCI and PD-MCI populations in the dCDT, which distinguished AD-MCI from PD-MCI patients, the area under the roc curve (AUC) = 0.923, 95% confidence interval (CI) = 0.866–0.983. The AUC for effective differentiation between AD-MCI and PD-MCI patients with high education (≥12 years of education) was 0.968, CI = 0.927–1.000. By correlation analysis, we found that the overall plotting of task performance score (*VFDB*_1_) correlated with the [visuospatial/executive] subtest score on the Montreal Cognitive Assessment (MoCA) scale (Spearman rank correlation coefficient [R] = 0.472, *p* < 0.001).

**Conclusion:**

The dCDT is a tool that can rapidly and accurately characterize and quantify differences in cognitive functioning in AD-MCI and PD-MCI populations.

## 1 Introduction

Mild cognitive impairment (MCI) is defined as a progressive decline in memory or other cognitive functions, while individuals with MCI are still able to maintain daily functioning. MCI is commonly seen in two neurodegenerative diseases, Alzheimer’s disease (AD) and Parkinson’s disease (PD) (Arvanitakis et al., 2019). According to the latest data, there are about 416 million people in the continuous spectrum of AD worldwide, 32 million of whom suffer from dementia (Gustavsson et al., 2023). AD can be divided into three stages of disease progression, namely preclinical AD, AD-derived mild cognitive impairment (AD-MCI), and AD dementia, of which AD-MCI is an important window for its early recognition (Knopman et al., 2021). However, the number of people with PD is currently over 10 million worldwide, and most of these patients develop cognitive dysfunction as the disease progresses (Wang et al., 2021). Studies have shown that half of newly diagnosed PD patients are associated with mild cognitive impairment after 3 years, and the conversion rate of mild cognitive impairment in PD (PD-MCI) patients to PD dementia (PDD) is close to 40% (Pedersen et al., 2017). Both AD-MCI and PD-MCI populations suffered from cognitive deficits. Compared to AD-MCI populations, PD-MCI populations had less severe memory deficits but more severe impairments in executive functioning, visuospatial ability, and attention (Brandão et al., 2020; Aamodt et al., 2021; Aarsland et al., 2021; Chandler et al., 2021). So, further fine-grained quantification of the differences in cognitive functioning between the two populations would help physicians more accurately diagnose the type of cognitive impairment in their patients and formulate targeted treatment plans.

At present, neuropsychological scales are mainly used to examine cognitive deficits in AD-MCI and PD-MCI populations, but they are highly participatory, time-consuming, and require clinician involvement (Lawson et al., 2021; Schmitter-Edgecombe et al., 2022; Conca et al., 2024). Scholars believed that digital biomarkers could be used to objectively characterize cognitive deficits in AD-MCI and PD-MCI populations at a fine-grained level (Ding et al., 2022; Park and Schott, 2022). Digital biomarkers are the use of digital averages to transform the “signals” emitted by humans into a quantifiable, clinically average and objective standard that can detect or predict disease progression (Coravos et al., 2019; Avram et al., 2020). Most importantly, they provided simpler and less costly continuity of real data and early detection of subtle changes (Dorsey et al., 2017; Gold et al., 2018). Therefore, the use of digital assessment is expected to quantify and characterize the differences in cognitive functioning between AD-MCI and PD-MCI populations at a fine-grained level, as well as provide a favorable reference for further accurate diagnosis of the types of cognitive impairment in AD-MCI populations and PD-MCI.

The clock drawing test (CDT) is a multidimensional cognitive functioning assessment tool that captures several aspects of cognitive functioning, such as executive functioning, planning, visuospatial ability, memory and attention (Dion et al., 2021). Whereas, the digital clock drawing test (dCDT) provides a more nuanced assessment of cognitive functioning status by capturing more detailed parameters. Schejter-Margalit et al. (2021) demonstrated that the use of a quantitative digital clock drawing test demonstrated greater sensitivity in identifying subtle cognitive declines in early Parkinson’s disease when compared to current standardized tests. Li et al.’s (2023) previous study showed that the digital clock mapping test could assess cognitive dysfunction at a fine-grained level in a population with MCI of AD origin and had good early warning efficacy. A Meta-analysis showed that the diagnostic performance of the digital clock drawing test was superior to that of the traditional pen-and-paper CDT as well as other types of digital drawing tests in AD-MCI populations (Chan et al., 2022). In addition, studies had been conducted to differentiate AD-MCI populations from PD-MCI populations based on clock-drawing test performance, and the results suggested that clock-drawing test could be used as a complementary tool to clinical diagnostic criteria for differentiating AD-MCI populations from PD-MCI populations (Saka and Elibol, 2009; Saur et al., 2012; Stagge et al., 2024). Studies on the application of the clock drawing test in comparing AD populations with cognitively impaired PD populations are detailed in Table 1.

**Table 1:** Application of the clock drawing test in comparing AD and PD cognitively impaired populations.

Researcher	Method	Limitation
Saka and Elibol (2009)	Participants draw watches on white paper. Points are awarded based on the result of the drawing of the clock.	(1) The sample sizes of AD-MCI patients and PD-MCI patients were small; (2) the dimensions of the extracted metrics were limited, and only the final drawn clock images were analyzed; and (3) the accuracy of distinguishing between AD-MCI patients and PD-MCI patients was low, with an AUC of only 0.668.
Saur et al. (2012)	Participants take a clock drawing test. Points are awarded based on the picture of the clock results drawn.	(1) The sample sizes of AD-MCI patients and PD cognitively impaired patients were small; (2) the dimensionality of the extracted metrics was limited and only analyzed on the final drawn clock pictures.
Allone et al. (2018)	Participants drew clocks on paper. The clock drawings were rated both quantitatively and qualitatively.	(1) The sample size of PD-MCI patients was small; (2) the dimensionality of metrics extraction was limited, and only the final drawn clock pictures were analyzed.
Jalakas et al. (2019)	Participants were given a clock drawing test. Scoring was based on pictures of the clock drawing results.	(1) There was a large difference in sample size ratios between AD and PDD patients; (2) performance on the clock-drawing test was compared between AD and PDD patients, but no significant differences were found; and (3) the dimensionality of the extracted metrics was limited, and only the final clock drawings were analyzed.
Tafiadis et al. (2021)	Participants were given a clock drawing test. Scoring was based on pictures of the clock drawing results.	(1) The sample sizes of patients with AD and PDD were small; (2) the performance of patients with AD and PDD on the clock-drawing test was compared, but no significant differences were found; and (3) the dimensionality of the extracted metrics was limited, and only the final clock drawings were analyzed.

Alzheimer’s disease (AD), Parkinson’s disease (PD), Mild cognitive impairment due to Alzheimer’s disease (AD-MCI), Parkinson’s disease with Mild cognitive impairment (PD-MCI), Parkinson’s disease with normal cognition (PD-NC), Parkinson’s disease dementia (PDD), area under the roc curve (AUC).

Most studies on clock drawing tests have focused on extracting metrics from the final clock drawing results, without a detailed analysis of the drawing process. This made it difficult to quantify fine-grained differences in cognitive functioning between AD-MCI and PD-MCI populations. For example, Jalakas et al. (2019) conducted a study that failed to find significant differences between AD and PDD populations in clock mapping tests. In contrast, the dynamic digital biomarker-based clock mapping method provided the possibility of objectively and accurately detecting differences in cognitive function between AD-MCI and PD-MCI populations, owing to its ability to quantify the entire clock mapping process at a fine-grained and continuous level.

In summary, we proposed the following research hypothesis: there is a difference in cognitive function between AD-MCI and PD-MCI populations in the digital clock drawing test, and the two populations can be effectively differentiated based on this feature. To test the hypothesis, we designed the dCDT, extracted digital biomarkers that can characterize cognitive function differences between AD-MCI and PD-MCI populations, and provided favorable references for the early diagnosis, treatment, and prevention of dementia progression in AD-MCI and PD-MCI populations.

## 2 Materials and methods

### 2.1 Participants recruitment

#### 2.1.1 Sample size estimation

We used the G*Power tool to approximate the final sample size for inclusion, with the relevant parameters being Test family: “F tests,” Statistical test: “ANOVA: Fixed effects, omnibus, one-way,” Type of power analysis: “*A priori*: Compute required sample size—given *α*, power, and effect size,” Effect size *f* = 0.3, α err prob = 0.05, Power (1–*β* err prob) = 0.9, Number of groups = 4, and the total sample size was calculated to be 164, i.e., 41 people were required for each of the NC group, AD-MCI group, PD-MCI group, and PD-NC group.

#### 2.1.2 Participant recruitment process and inclusion criteria

In this study, 175 participants were recruited from the Department of Neurology and the Department of Nuclear Medicine of the Second Medical Center of the General Hospital of the Chinese People’s Liberation Army. A total of 165 participants were followed up in the trial, including 41 patients with AD-MCI, 42 patients with PD-MCI, 42 patients with PD-NC, and 40 NC controls. During the formal trial, one AD-MCI patient withdrew due to disease progression, and two PD-MCI patients and one PD-NC patient could not participate for unspecified reasons. This left an effective sample size of 161, including 40 AD-MCI patients, 40 PD-MCI patients, 41 PD-NC patients, and 40 normal controls. PD-MCI and PD-NC patients were discontinued within 12 h prior to dCDT. The participant screening process is shown in Supplementary Figure 1. The demographic characteristics of the participants is shown in Supplementary Table 1. To ensure consistency of data, all participants completed the dCDT, MMSE, and MoCA sequentially on the same day. The MDS-UPDRS assessment was also completed by all participants except those in the AD-MCI group.

All of the above participants were native speakers of Chinese and given a definite diagnosis by clinical experts. Participants’ general information data included age, gender, years of education, Minimum Mental State Examination (MMSE) score, Montreal Cognitive Assessment (MoCA) score, and Movement Disorder Society Unified Parkinson’s Disease Rating Scale III (MDS-UPDRS III) score. The above MMSE, MoCA, and MDS-UPDRSIII scales are all standardized Chinese versions (Yu et al., 2017; Jia et al., 2021). All experimental procedures were in accordance with the Helsinki Declaration and approved by the Medical Ethics Committee of the Chinese People’s Liberation Army General Hospital (Ethics No. S2022-770-02). Eligible participants were collected according to the following inclusion and exclusion criteria.

AD-MCI patients inclusion criteria: (1) met clinical MCI diagnostic criteria developed by the National Institute on Aging (NIA) and Alzheimer’s Association (ADA) in 2011; (2) 11C-PIB PET/CT positive imaging; (3) the dominant hand was the right hand and was able to cooperate in completing test; (4) aged 45–80 years old, gender was not limited; and (5) signed informed consent form.

PD-MCI patients inclusion criteria: (1) met the British Brain Bank PD diagnostic criteria; (2) Parkinson’s disease background, by the patient’s family statement or clinician found that the patient’s gradual cognitive decline; (3) neuropsychological test cognitive impairment; (4) cognitive impairment, but had not yet significantly intervened in the patient’s functional independence; (5) the affected side or the more serious are the right side of the limb, the habitual hand for the right hand and able to cooperate in the completion of the test; (6) aged 45–80 years old, gender was not limited; and (7) signed informed consent form.

PD-NC patients inclusion criteria: (1) met the diagnostic criteria of the British Brain Bank for PD; (2) cognitive decline was not observed by patient informants or clinicians; (3) cognitive decline was not reflected in neuropsychological tests or overall cognitive scales; (4) affected side or more severely all right limb, dominant hand was right hand, and they were able to cooperate with completion of test; (5) aged 45–80 years old, gender was not limited; and (6) signed informed consent form.

NC inclusion criteria: (1) no complaints and objective evidence of neurologic disease (normal neurologic clinical examination); (2) no cognitive impairment; (3) habitual hand is right-handed and able to cooperate with the test; (4) aged 45–80 years old, gender was not limited; and (5) signed informed consent form.

Exclusion criteria for all participants: (1) history of schizophrenia, severe anxiety and depression, and other psychiatric disorders; (2) history of severe head injury and other serious illnesses; (3) history of alcohol and drug abuse; and (4) other conditions that may prevent completion of the test (including arm disability, etc.).

### 2.2 Design of digital clock drawing test and digital biomarkers

Based on the research hypothesis that there is a difference in cognitive functioning between AD-MCI and PD-MCI populations in the dCDT, and that this feature is effective in distinguishing between these two populations, we designed the dCDT using projected intercapacitive haptic feedback technology. Details of the test are outlined below.

#### 2.2.1 Experimental test design prerequisite

**Figure 1:**
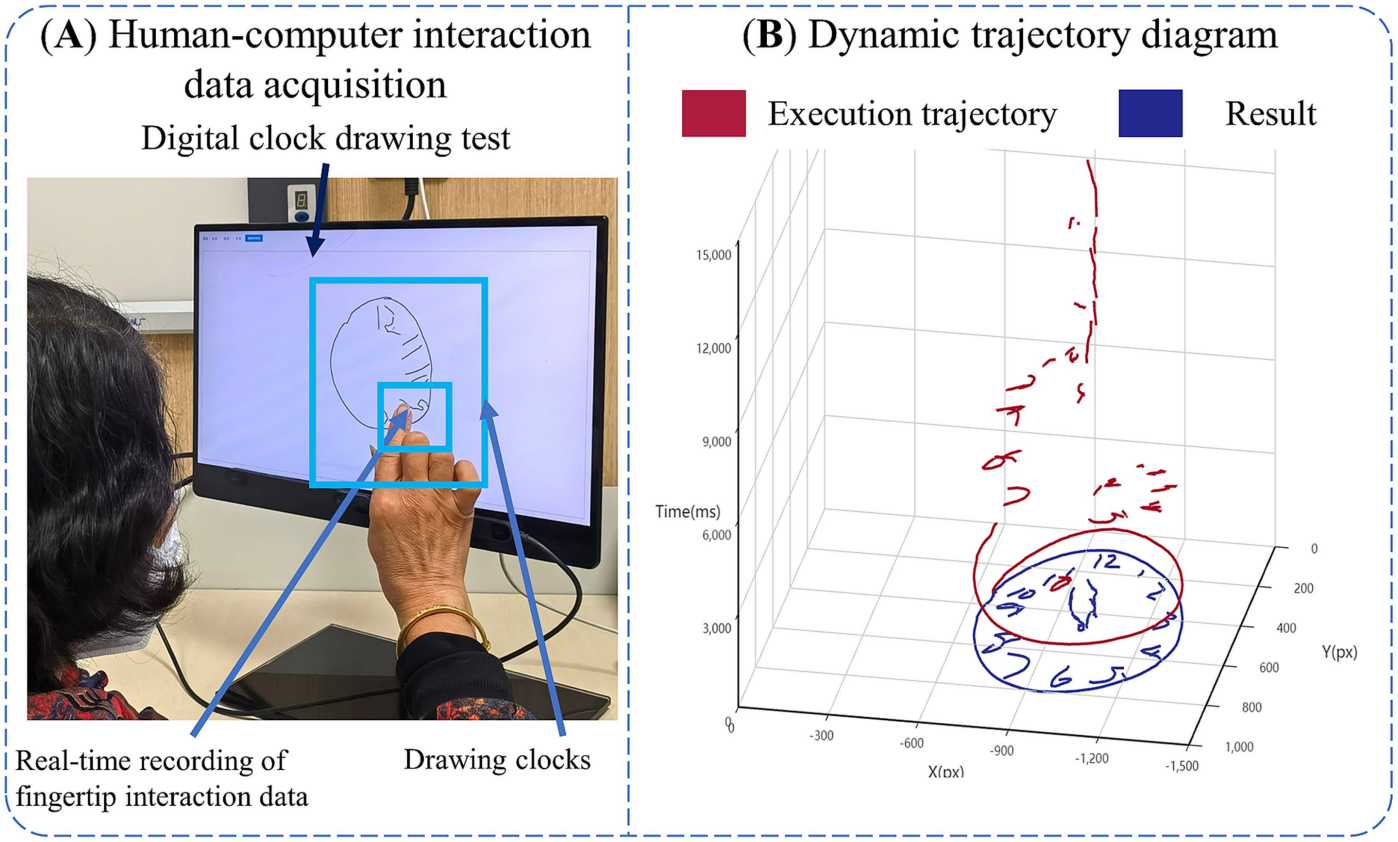
Introduction of digital clock drawing testing. **(A)** Human-computer interaction data acquisition. **(B)** Dynamic trajectory diagram.

The hardware required for this experiment consists of an Intel computer (NUC11PAHi5), a touchable monitor with 3,840 × 2,160 pixels (Length, width and height 392 × 250 × 10 mm, screen size 17.3 inches). The software system involved in this experiment is a human-computer interaction system. We built the front-end interface of this system through Electron and Vue3, and constructed dCDT through HTML5 Canvas, and the sampling frequency of human-computer interaction data in the test assessment process was about 55 Hz. We built the back-end system of this system through python, and built the human-computer interaction database through Mysql database. Human-computer interaction data acquisition is shown in Figure 1A.

#### 2.2.2 Experimental test design and principle interpretation

The clock drawing test can be used as a cognitive function assessment tool that involves the synergistic effect of multidimensional cognitive functions such as executive function and visuospatial function. We designed the dCDT to quantify the entire clock-drawing process, collect real-time human-computer interaction data reflecting participants’ visuospatial and executive functions, and then evaluate participants’ cognitive functions in the test process.

The target of the dCDT was that participants need to draw a clock at 11:10 on the screen with their right index finger through fingertip interaction, and they need to write down all the digits and clock hands on the clock face, and the test is limited to 3 min.

#### 2.2.3 Definition and quantitative analysis of digital biomarkers

We extracted digital biomarkers from the database via python (3.10.0) based on the above objectivized human-computer interaction data. To compare cognitive functioning differences between AD-MCI and PD-MCI populations in the dCDT at a fine-grained level, we classified digital biomarkers into visuospatial function digital biomarkers and executive function digital biomarkers.

The visuospatial function digital biomarkers (*VFDB*) were used to reflect participants’ ability to process, understand, and respond in the visuospatial environment of a painted clock, and to assess participants’ ability to translate the visual image of a clock (clock numbers, outline, and clock hands) into a concrete concept of time or mathematical representation, and to focus on clock numbers, outline, and hand positions on the clock dial, as well as to effectively ignore other irrelevant visual information. The *VFDB* was scored on the participant’s image of the clock-drawing result, including an overall score on the participant’s image of the clock (Task Performance of Overall Drawing Score), individual scores on the outline of the clock (Task Performance of Outline Drawing Score), individual scores on the numbers within the clock (Task Performance of Numbers Drawing Score), and individual scores on the clock hands (Task Performance of Clock Hands Drawing Score).

The executive function digital biomarkers (*EFDB*) were designed to reflect the participant’s ability to plan, strategize, and solve problems in the dCDT. The *EFDB* was measured using a fingertip interaction technique to assess participants’ planning, conceptualization, and recall of the clock drawing prior to “drawing execution,” including Task Completion Time, Total Drawing Pause Time, Initial Drawing Pause Time, Drawing Process Pause Time (including total time, average time, and maximum time) and Number of Pauses during Drawing. Task Completion Time was used to describe the time it took participants to draw the complete clock. Total Drawing Pause Time was used to describe the overall thinking and planning of participants during clock drawing. Initial Drawing Pause Time was used to describe participants’ planning and thinking before the clock was drawn. Drawing process Pause Time and Number of Pauses during Drawing. They were used to describe participants’ planning and thinking about the details of the clock drawing during the assessment process.

At the same time, the *EFDB* was also designed to reflect participants’ drawing performance after “planning” through fingertip interaction technology, including Drawing Time, Number of Draws, Efficiency of Drawing, Length of Drawn Line, Initial Drawing Speed, Average of Drawing Speed, Variability of Drawing Speed. Drawing Time was used to describe the time participants spent drawing the clock. Number of Draws was used to describe the number of strokes made by the participants in the clock drawing. Efficiency of Drawing was used to describe how efficiently participants drew the clock. Length of Drawing Line was used to describe the length of lines drawn by participants during the clock drawing process. Initial drawing speed, Average of drawing speed, Variability of drawing speed were used to describe the magnitude and degree of variability of participants’ drawing speed during the clock drawing process.

To facilitate future digital biomarker mining analyses, we provided a detailed conceptual definition of the various digital biomarkers in the test:

(1) Visuospatial Function Digital Biomarkers (*VFDB*)

**Figure 2:**
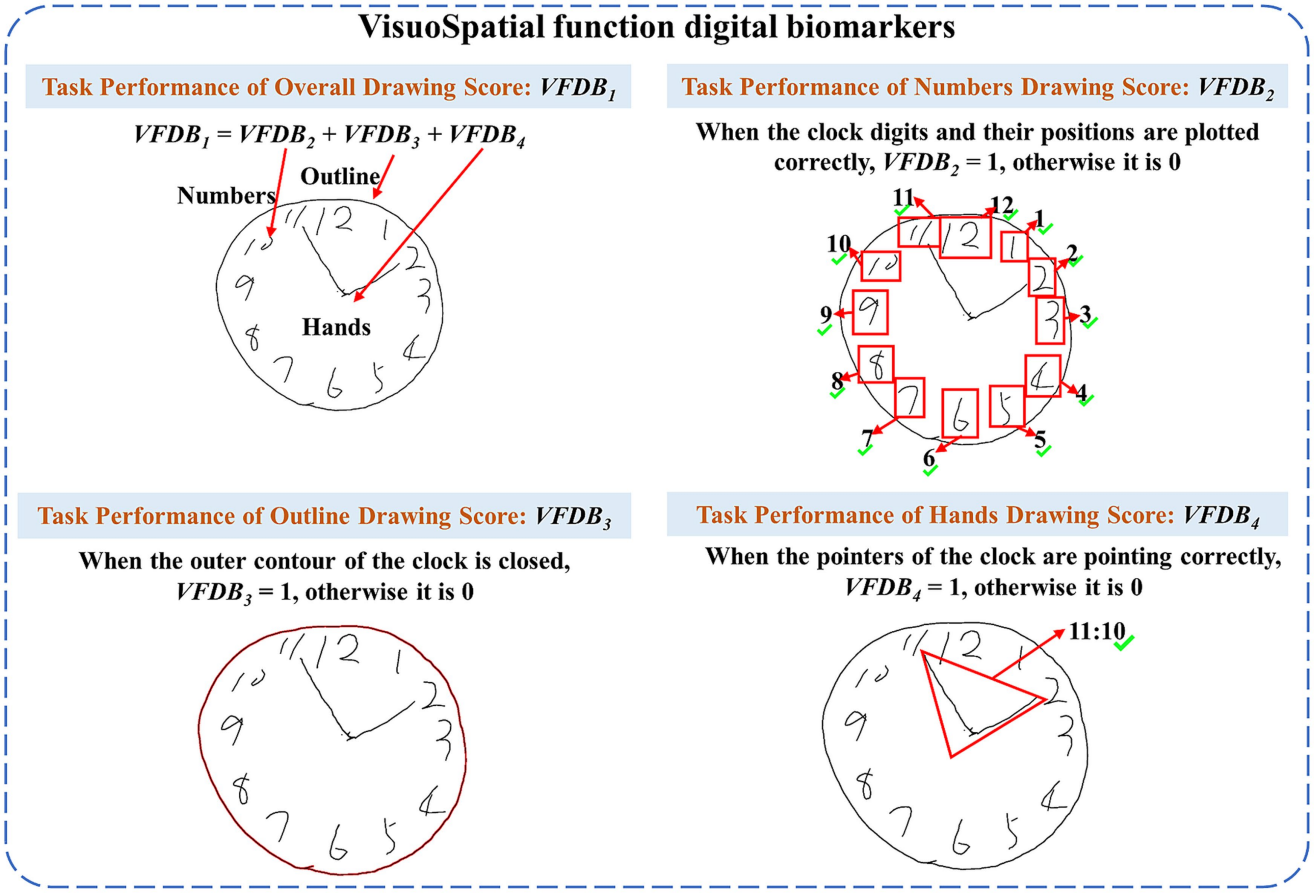
Graphical representation of visuospatial function digital biomarkers.

Descriptions of visuospatial function digital biomarkers are shown in Table 2, and a graphical representation of digital visuospatial function biomarkers is shown in Figure 2.

(2) Executive Function Digital Biomarkers (EFDB)

**Table 2:** Descriptions of the visuospatial function digital biomarkers.

Cognitive impairment	Digital biomarkers	Abbreviation	Interpretation and units
Patients with AD-MCI and PD-MCI exhibit impairments in visuospatial function (North et al., 2021; Hannaway et al., 2023)	Visuospatial function digital biomarker_1_: Task Performance of Overall Drawing Score	*VFDB*_1_	It was obtained by calculating the sum of Task Performance of Numbers Drawing Score, Task Performance of outline Drawing Score, Task Performance of Clock Hands Drawing Score by the participant, and then obtaining the participant’s overall drawing score (Unit: points).
	Visuospatial function digital biomarker_2_: Task Performance of Numbers Drawing Score	*VFDB*_2_	It was used to determine whether the participant drew the numbers in the clock and correctly placed the numbers, and to calculate the participant’s drawing score (Unit: points).
	Visuospatial function digital biomarker_3_: Task Performance of outline Drawing Score	*VFDB*_3_	It was used to determine whether the outline of a clock drawn by a participant is closed or not, and to calculate the participant’s outline-drawing score (Unit: points).
	Visuospatial function digital biomarker_4_: Task Performance of Clock Hands Drawing Score	*VFDB*_4_	It was used to assess whether the clock hands were correctly indicating the hour and minutes, and to calculate the participant’s clock hands drawing score (Unit: points).

**Figure 3:**
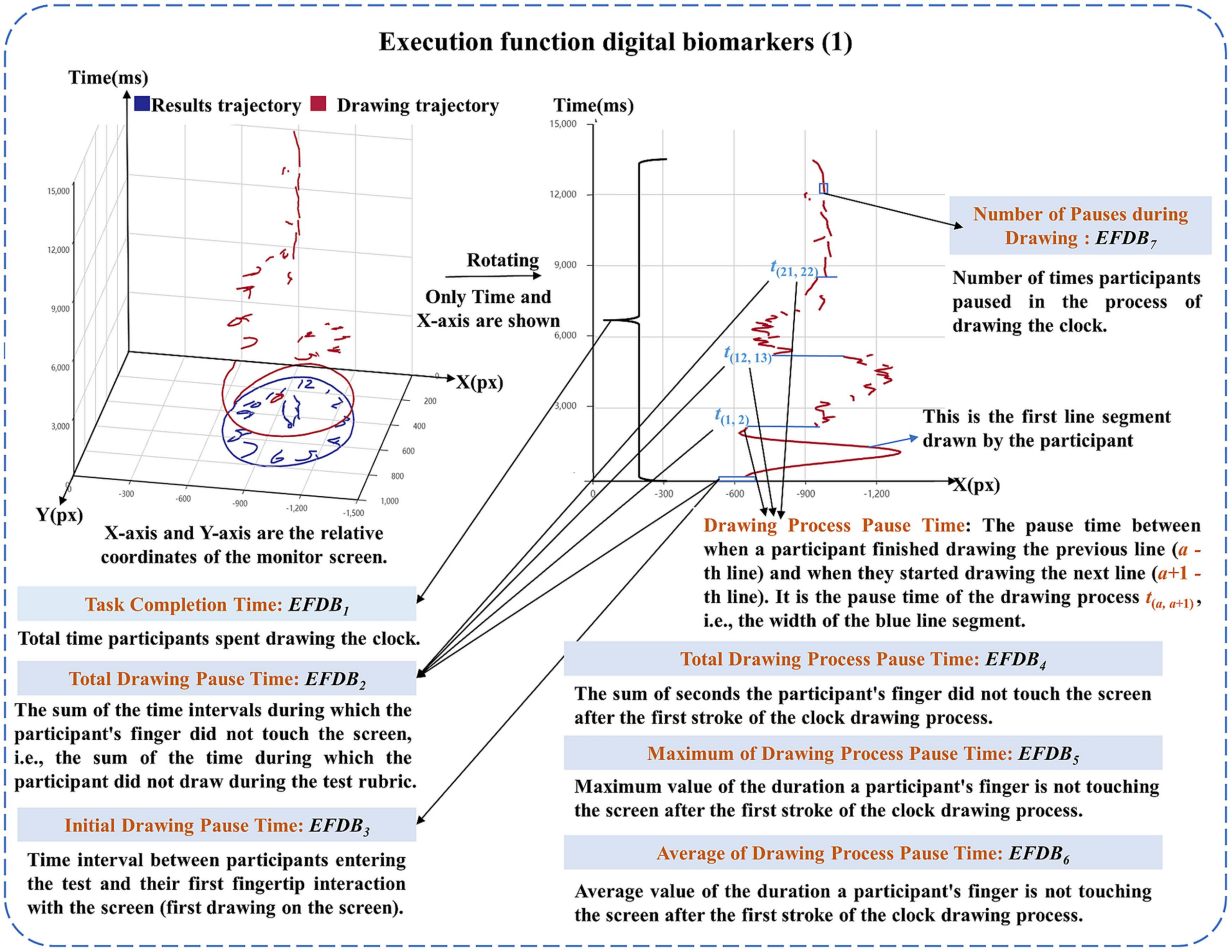
Graphical representation of the executive function digital biomarkers (1).

**Figure 4:**
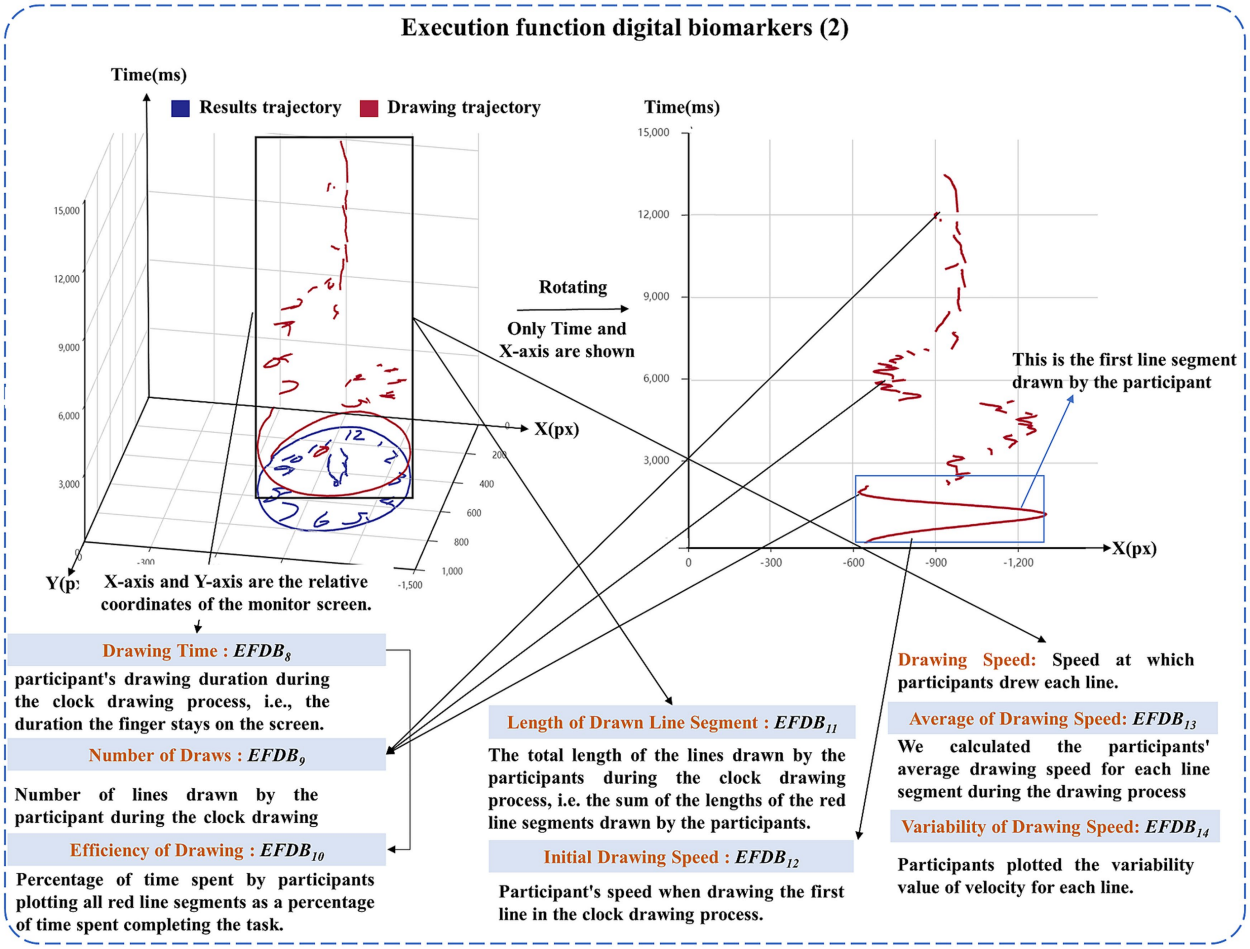
Graphical representation of the executive function digital biomarkers (2).

Descriptions of the executive function digital biomarkers are shown in Table 3, and a graphical representation of the executive function digital biomarkers is shown in Figures 3, 4.

**Table 3:** Descriptions of the executive function digital biomarkers.

Cognitive impairment	Digital biomarkers	Abbreviation	Interpretation and units
Patients with AD-MCI and PD-MCI exhibit impairments in executive function (Arrigoni et al., 2024; Zhang et al., 2024)	Executive function digital biomarker_1_: Task Completion Time	*EFDB*_1_	It was used to calculate the time taken by the participant from entering the test to completing the clock drawing, i.e., the total time taken to complete the test (Unit: seconds, s).
	Executive function digital biomarker_2_: Total Drawing Pause Time	*EFDB*_2_	It was used to calculate the sum of the time intervals during which the participant’s finger did not touch the screen, i.e., the sum of the time during which the drawing was not performed, during the test (Unit: seconds, s).
	Executive function digital biomarker_3_: Initial Drawing Pause Time	*EFDB*_3_	It was used to calculate the time between a participant’s entry into the test and the first drawing on the screen (Unit: seconds, s).
	Executive function digital biomarker_4_: Total Drawing Process Pause Time	*EFDB*_4_	It was used to calculate the sum of the time the participant’s finger did not touch the screen after the first stroke was drawn during the clock drawing process (Unit: seconds, s).
	Executive function digital biomarker_5_: Maximum of Drawing Process Pause Time	*EFDB*_5_	It was used to calculate the maximum value of the duration that a participant’s finger does not touch the screen after the first stroke of the clock drawing process (Unit: seconds, s).
	Executive function digital biomarker_6_: Average of Drawing Process Pause Time	*EFDB*_6_	It was used to calculate the average duration that a participant’s finger does not touch the screen after the first stroke during the clock drawing process (Unit: seconds, s).
	Executive function digital biomarker_7_: Number of Pauses during Drawing	*EFDB*_7_	It was used to count the number of times a participant’s finger stays on the screen during the clock drawing process (Unit: times).
	Executive function digital biomarker_8_: Drawing Time	*EFDB*_8_	It was used to calculate total time the participant actively spent drawing, i.e., the duration the finger stays on the screen (Unit: seconds, s).
	Executive function digital biomarker_9_: Number of Draws	*EFDB*_9_	It was used to count the number of times a participant drew a line during the clock drawing process. (Unit: times).
	Executive function digital biomarker_10_: Efficiency of Drawing	*EFDB*_10_	It was used to calculate the drawing efficiency of the participants. The participant’s drawing time during the clock drawing process, i.e., the time the finger stays on the screen, is first calculated, and then analyzed as a percentage of the task completion time (the total elapsed time to complete the test), which is Drawing Efficiency (Unit: %).
	Executive function digital biomarker_11_: Length of Drawn Line	*EFDB*_11_	It was used to calculate the total length of the line drawn by the participant during the clock drawing process (Unit: pixels, px).
	Executive function digital biomarker_12_: Initial Drawing Speed	*EFDB*_12_	It was used to calculate the speed at which the participant drew the first line in the clock drawing process (Unit: pixels/seconds, px/s).
	Executive function digital biomarker_13_: Average of Drawing Speed	*EFDB*_13_	It was used to calculate the average speed at which participants drew each line during the clock drawing process (Unit: pixels/seconds, px/s).
	Executive function digital biomarker_14_: Variability of Drawing Speed	*EFDB*_14_	It was used to calculate the variability of a participant’s drawing speed. That is, the degree of variability in the speed at which the participant draws each line is calculated during the clock drawing process (Unit: %).

The algorithm for analyzing the above digital biomarkers is as follows:

We built on pre-existing algorithms (Li et al., 2023), to obtain Task Performance of Overall Drawing Score (*VFDB*_1_), Task Performance of Numbers Drawing Score (*VFDB*_2_), Task Performance of Outline Drawing Score (*VFDB*_3_) and Task Performance of Clock Hands Drawing Score (*VFDB*_4_), Task Performance of Overall Drawing Score (*VFDB*_1_) was calculated by Equation 1:


(1) $$VFDB_{1}=VFDB_{2}+VFDB_{3}+VFDB_{4}$$

We applied Optical Character Recognition to calculate the Task Performance of Numbers Drawing Score (*VFDB*_2_), assigning a score of 1 if the clock digits were complete and distributed clockwise, and 0 otherwise. We applied contour edge detection to calculate the Task Performance of Outline Drawing Score (*VFDB*_3_), assigning a score of 1 if the outer contour of the clock was closed, and 0 otherwise. We computed the Task Performance of Clock Hands Drawing Score (*VFDB*_4_) using the Spatial Transformer Network clock recognition architecture, if the clock time was recognized as 11:10, the Task Performance of Clock Hands Drawing Score (*VFDB*_4_) was 1, otherwise, it was 0. The clock time was recognized by the equation:


(2) $$T=\Phi \left(\text{Pictures}\right)\in P^{720}$$

In Equation 2, *T* was the time of the predicted clock, *Pictures* was the clock image drawn by the participants, and *Φ* was the classification network. There will be 12 results for hours, i.e., 1:00 to 12:00; and 60 results for minutes, i.e., 0 to 59:00. There were 720 ways to combine time. *P*^720^ was 720 classification results, and the calculation of the task. The calculation of Task Performance Calculation of Hand Drawing Score (*VFDB*_4_) is shown in Supplementary Figure 2.

We recorded the participant’s Task Completion Time (*EFDB*_1_) and Initial Drawing Pause Time (*EFDB*_3_). Let the participant’s Number of Draws (*EFDB*_9_) was *A*. For each draw: The *a*-th drawn line (1 ≤ *a* ≤ *A*, *a* ∈ N) was labeled *L_a_*. The drawing duration for the *a*-th line was *t_a_*. Line *L_a_* consisted of *B_a_* drawing coordinate points, with the *b*-th drawing coordinate point (1 ≤ *b* ≤ *B_a_*, *b* ∈ N) denoted as ($X_{b}$, $Y_{b}$). The pause time between the completion of line *L_a_* and the start of line *L_a_* + 1 was *t*_(*a*, *a* + 1)_, Total Drawing Pause Time (*EFDB*_2_), Total Drawing Process Pause Time (*EFDB*_4_), Maximum of Drawing Process Pause Time (*EFDB*_5_), Average of Drawing Process Pause Time (*EFDB*_6_), Number of Pauses during Drawing (*EFDB*_7_) were calculated by Equations 3–8:


(3) $$EFDB_{2}=EFDB_{3}+EFDB_{4}$$

(4) $$EFDB_{4}=\sum_{a=1}^{A-1}t_{\left(a,a+1\right)}\left(1\leq a\leq A-1,a\in N\right)$$

(5) $$EFDB_{5}=\max_{1\leq a\leq A-1}t_{\left(a,a+1\right)}$$

(6) $$EFDB_{6}=\frac{EFDB_{4}}{A-1}$$

(7) $$D\left(b\right)=\{\;\begin{array}{c} 1\;\left(X_{b+1}=X_{b}\;\text{and}\;Y_{b+1}=Y_{b}\right)\\ 0\;\left(X_{b+1}\neq X_{b}\;\text{or}\;Y_{b+1}\neq Y_{b}\right) \end{array}$$

(8) $$EFDB_{7}=\sum_{a=1}^{A}\sum_{b=1}^{B_{a}-1}D\left(b\right)$$

In Equation 7, $\sum_{b=1}^{B_{a}-1}D\left(b\right)$ was used to calculate the number of pauses in judging a particular drawing of a line, and *A* indicated that the participant drew a total of *A* lines.

Drawing Time (*EFDB*_8_), Efficiency of Drawing (*EFDB*_10_), and Length of Drawn Line (*EFDB*_11_) were calculated by Equations 9–12:


(9) $$EFDB_{8}=\sum_{a=1}^{A}t_{a}\left(1\leq a\leq A,a\in N\right)$$

(10) $$EFDB_{10}=\frac{EFDB_{8}}{EFDB_{1}}$$

(11) $$L\left(L_{a}\right)=\sum_{b=1}^{B_{a}-1}\sqrt{{\left(X_{b+1}-X_{b}\right)}^{2}+{\left(Y_{b+1}-Y_{b}\right)}^{2}}$$

(12) $$EFDB_{11}=\sum_{a=1}^{A}L\left(L_{a}\right)$$

In Equation 9, $\sum_{b=1}^{B_{a}-1}\sqrt{{\left(X_{b+1}-X_{b}\right)}^{2}+{\left(Y_{b+1}-Y_{b}\right)}^{2}}$ was used to calculate the length of the line *L_a_* drawn by the participants.

We separately calculated the speed at which the participants draws each line, i.e., the speed *V_a_* at which the *a*-th line *L_a_* (which has a total of *B_a_* drawing coordinate points) was drawn, and then calculated the Initial Drawing Speed (*EFDB*_12_), Average of Drawing Speed (*EFDB*_13_) and Variability of Drawing Speed (*EFDB*_14_), which were given by Equations 13–17:


(13) $$V_{a}=\frac{L\left(L_{a}\right)}{t_{a}}\left(1\leq a\leq A,i\in N\right)$$

(14) $$EFDB_{12}=V_{1}$$

(15) $$EFDB_{13}=\sum_{a=1}^{A}\frac{V_{a}}{A}\left(1\leq a\leq A,i\in N\right)$$

(16) $$\sigma =\sqrt{\frac{\sum_{a=1}^{A}{\left(V_{a}-EFDB_{13}\right)}^{2}}{A}}\left(1\leq a\leq A,i\in N\right)$$

(17) $$EFDB_{14}=\frac{\sigma }{EFDB_{13}}$$

In Equation 16, σ was the standard deviation of the drawing speed when participants draw all lines during the test.

#### 2.2.4 Design of experimental rules

Participants were in a quiet room for the test assessment to prevent the results from being affected by the noisy environment. We positioned a comfortable and stable chair in front of the display with touch screen function, after the participant sat down, their posture was adjusted to maintain an approximate distance of 30 cm between their upper body and the display. This setup ensured clear visibility and comfortable finger-based interaction, minimizing potential visual interference or operational discomfort that could compromise the experimental results. In addition, all participants in this experiment were right-handed, and the affected side of PD-MCI patients and PD-NC patients were on the right side, so as not to interfere with the experimental results by hand habits.

### 2.3 Experimental settings

#### 2.3.1 Experimental procedures

Prior to the official launch of the dCDT, we trained the staff in advance, informing them of the testing process of the dCDT and the operation of the human-computer interaction system, and subsequently the experimenter will inform participants on the test process, objectives, and instructions. All participants were tested in a quiet room. We positioned a comfortable and stable chair in front of display with touch screen function. After the participants were seated, the experimenter assisted will adjust their posture to maintain an approximate distance of 30 cm between their upper body and the display. Participants were asked to draw a clock at 11:10 with the fingertips of their right index finger, and the test was limited to 3 min. If a participant took longer than 3 min to complete the task, they were deemed to have failed the protocol and were excluded from further analysis. The dynamic trajectory of the dCDT is shown in Figure 1B.

### 2.4 Statistical analysis

All statistical analyses were performed using the Universal Data Analysis Software SPSS 26.0 package. We conducted a comparative analysis of the data from the NC, AD-MCI, PD-MCI, and PD-NC groups. Count data were compared between groups using chi-square test. Measurement information conforming to the normal distribution was expressed as mean ± standard deviation (x ± s), and one-way Analysis of Variance (ANOVA) was used to compare the differences between multiple groups, and when there was a difference, the least significant difference (LSD) method was used for one-to-one comparisons used for pairwise comparisons between groups. Measurement data conforming to the skewed distribution were expressed as median (interquartile spacing), and the Kruskal-Wallis H test was used to compare the differences between multiple groups, and multiple comparisons between groups were performed. When differences existed, the Bonferroni method was used to compare each comparison group individually (differences were compared by corrected *p*-values). Additionally, we stratified the AD-MCI and PD-MCI groups by educational attainment and conducted comparative analyses specifically within the higher education subgroup (years of education ≥12 years). Continuous variables with normal distributions were presented as mean ± standard deviation (x ± s), and intergroup differences were compared using independent samples t-tests. For continuous variables with skewed distributions, data were expressed as median (interquartile spacing) and analyzed using Mann–Whitney U tests. Qualitative information was expressed as a rate (%). We used a binary logistic regression model to plot the receiver operating characteristic curve (ROC) and determined the accuracy of distinguishing between different populations for individual digital biomarkers and multiple combined digital biomarkers by comparing the area under the roc curve (AUC). In conducting correlation analyses between digital biomarkers and the Montreal Cognitive Assessment (MoCA) total scores with selected subdomain scores, Pearson linear correlation analysis was applied when both continuous variables exhibited normal distributions. For non-normally distributed variables, Spearman rank correlation analysis was utilized. *p* < 0.05 was considered to indicate a statistically significant difference.

## 3 Results

### 3.1 Demographic and clinical characteristics

The effective sample size of this study was 161, including 40 AD-MCI patients, 40 PD-MCI patients, 41 PD-NC patients, and 40 normal cognitive controls. They were included in the AD-MCI group, PD-MCI group, PD-NC group, and NC group, respectively. We analyzed the difference in baseline data of the four groups, including age, gender, years of education, MMSE, MOCA, and MDS-UPDRS III of the participants in the four groups, and the results of demographic difference analysis for each group are shown in Table 4.

**Table 4:** The results of demographic difference analysis for each group.

	NC (*n* = 40)	AD-MCI (*n* = 40)	PD-MCI (*n* = 40)	PD-NC (*n* = 41)	NC vs. AD-MCI	NC vs. PD-MCI	NC vs. PD-NC	AD-MCI vs. PD-MCI	PD-MCI vs. PD-NC	AD-MCI vs. PD-NC	NC vs. AD-MCI vs. PD-MCI vs. PD-NC
					Corrected *p* value						*p* value, df
Age, years	61.00 (15.25)	65.50 (13.00)	66.00 (12.00)	65.00 (9.00)	>0.05	>0.05	>0.05	>0.05	>0.05	>0.05	0.470, 3
Sex (female/male)	22/18	21/19	21/19	17/24	>0.05	>0.05	>0.05	>0.05	>0.05	>0.05	0.617, 3
Years of education	12.00 (6.00)	12.00 (6.00)	12.00 (0.00)	12.00 (3.50)	>0.05	>0.05	>0.05	>0.05	>0.05	>0.05	0.942, 3
MMSE	29.00 (2.00)	25.50 (2.00)	26.00 (2.75)	28.00 (1.50)	**<0.001**	**<0.001**	0.425	1.000	**<0.001**	**<0.001**	**<0.001**, 3
MoCA	25.00 (3.00)	22.00 (3.00)	21.00 (4.75)	25.00 (1.00)	**<0.001**	**<0.001**	1.000	1.000	**<0.001**	**<0.001**	**<0.001**, 3
MDS-UPDRS III	4.00 (2.00)	/	19.50 (12.75)	16.00 (8.00)	/	**<0.001**	**<0.001**	/	1.000	/	/

Because there were no MDS-UPDRS III data for AD-MCI, the NC, PD-MCI, and PD-NC groups were compared in the comparison of differences between multiple groups (*p* < 0.001, df = 2). Highlighting significant *p*-values (*p* < 0.05) in bold.

Normal cognition (NC), Mild cognitive impairment due to Alzheimer’s disease (AD-MCI), Parkinson’s disease with Mild cognitive impairment (PD-MCI), Parkinson’s disease with normal cognition (PD-NC), *p*-values (p), degree of freedom (df), Minimum Mental State Examination (MMSE), Montreal Cognitive Assessment (MoCA), Movement Disorder Society Unified Parkinson’s Disease Rating Scale III (MDS-UPDRS III).

In the NC, AD-MCI, PD-MCI and PD-NC groups, there were no statistical differences in age (*p* = 0.470, degree of freedom [df] = 3), gender (*p* = 0.617, df = 3), or years of education (*p* = 0.942, df = 3). However, there were statistical differences in MMSE (*p* < 0.001, df = 3) and MoCA (*p* < 0.001, df = 3). In the NC and AD-MCI groups, there were statistical differences in MMSE (*p* < 0.001) and MoCA (*p* < 0.001). In the NC and PD-MCI groups, there were statistical differences in MMSE (*p* < 0.001), MoCA (*p* < 0.001) and MDS-UPDRS III (*p* < 0.001). In the NC and PD-NC groups, there were no statistical differences in MMSE (*p* = 0.425) and MoCA (*p* = 1.000). There were statistical differences in MDS-UPDRS III (*p* < 0.001). In the AD-MCI and PD-MCI groups, there were no statistical differences in MMSE (*p* = 1.000) and MoCA (*p* = 1.000). In the PD-MCI and PD-NC groups, there were statistical differences in MMSE (*p* < 0.001) and MoCA (*p* < 0.001). There were no statistical differences in MDS-UPDRS III (*p* = 1.000). In the AD-MCI and PD-NC groups, there were statistical differences in MMSE (*p* < 0.001), MoCA (*p* < 0.001).

### 3.2 Analysis of digital biomarkers

We analyzed the differences in digital biomarkers in the NC, AD-MCI, PD-MCI and PD-NC groups. The results of the differential analysis of digital biomarkers for each group are shown in Table 5.

**Table 5:** The results of the differential analysis of digital biomarkers for each group.

	NC (*n* = 40)	AD-MCI (*n* = 40)	PD-MCI (*n* = 40)	PD-NC (*n* = 41)	NC vs. AD-MCI	NC vs. PD-MCI	NC vs. PD-NC	AD-MCI vs. PD-MCI	PD-MCI vs. PD-NC	AD-MCI vs. PD-NC	NC vs. AD-MCI vs. PD-MCI vs. PD-NC
					Corrected *p* value						*p* value, df, F*
Visuospatial function digital biomarkers											
*VFDB*_1_	3.00 (1.00)	2.00 (1.00)	2.00 (1.75)	2.00 (1.50)	**<0.001**	**0.001**	**0.004**	1.000	1.000	1.000	**<0.001**, 3
*VFDB*_2_	1.00 (0.00)	1.00 (1.00)	1.00 (1.00)	1.00 (1.00)	**0.002**	**0.012**	0.069	1.000	1.000	1.000	**0.002**, 3
*VFDB*_3_	1.00 (1.00)	0.50 (1.00)	1.00 (1.00)	0.00 (1.00)	>0.05	>0.05	>0.05	>0.05	>0.05	>0.05	0.244
*VFDB*_4_	1.00 (0.00)	0.50 (1.00)	0.00 (1.00)	1.00 (1.00)	**0.019**	**0.009**	0.096	1.000	1.000	1.000	**0.005**, 3
Executive function digital biomarkers											
*EFDB*_1_	39.61 (19.48)	44.64 (34.12)	75.92 (43.03)	50.33 (24.33)	0.789	**<0.001**	0.056	**<0.001**	**0.002**	1.000	**<0.001**, 3
*EFDB*_2_	22.82 (15.34)	28.52 (27.84)	50.02 (38.63)	30.02 (20.43)	0.328	**<0.001**	0.141	**0.001**	**0.002**	1.000	**<0.001**, 3
*EFDB*_3_	3.61 (2.74)	3.30 (3.35)	5.95 (5.82)	6.08 (6.69)	1.000	0.096	0.491	0.056	1.000	0.317	**0.019**, 3
*EFDB*_4_	19.52 (14.39)	24.54 (27.67)	45.49 (38.53)	22.33 (14.96)	0.224	**<0.001**	1.000	**0.003**	**<0.001**	1.000	**<0.001**, 3
*EFDB*_5_	4.21 (5.80)	5.11 (9.30)	12.50 (15.59)	5.14 (4.81)	0.781	**<0.001**	1.000	**0.034**	**0.016**	1.000	**<0.001**, 3
*EFDB*_6_	0.79 (0.61)	0.87 (0.61)	1.44 (1.20)	0.87 (0.45)	1.000	**<0.001**	1.000	**0.001**	**<0.001**	1.000	**<0.001**, 3
*EFDB*_7_	43.00 (35.00)	44.50 (64.00)	76.00 (87.00)	72.00 (199.00)	1.000	**<0.001**	**0.006**	**0.030**	1.000	0.356	**<0.001**, 3
*EFDB*_8_	16.42 (7.44)	13.92 (7.23)	22.79 (9.06)	17.64 (8.60)	1.000	**<0.001**	0.201	**<0.001**	0.148	**0.005**	**<0.001**, 3
*EFDB*_9_	24.00 (5.75)	26.50 (10.75)	28.00 (12.00)	26.00 (6.50)	0.243	**0.028**	1.000	1.000	0.460	1.000	**0.029**, 3
*EFDB*_10_	0.40 ± 0.10	0.33 ± 0.12	0.32 ± 0.10	0.39 ± 0.12	**0.005**	**0.001**	0.570	0.662	**0.007**	**0.023**	**0.002**, 3, 5.358
*EFDB*_11_	4702.75 (1796.29)	5145.90 (1414.18)	5323.38 (1638.93)	5419.64 (1169.04)	>0.05	>0.05	>0.05	>0.05	>0.05	>0.05	0.196
*EFDB*_12_	756.60 ± 306.98	867.75 ± 396.20	532.79 ± 274.22	610.86 ± 327.57	0.133	**0.003**	**0.048**	**<0.001**	0.288	**0.001**	**<0.001**, 3, 8.258
*EFDB*_13_	268.05 (140.69)	307.53 (184.96)	171.28 (66.20)	207.13 (131.64)	1.000	**<0.001**	0.737	**<0.001**	**0.017**	**0.025**	**<0.001**, 3
*EFDB*_14_	71.71 (85.05)	118.16 (64.06)	92.10 (71.93)	91.91 (69.28)	**0.004**	1.000	1.000	**0.034**	1.000	**0.030**	**0.003**, 3

* means that when a numerical biomarker satisfies a normal distribution, its *F*-value will be listed. Highlighting significant *p*-values (*p* < 0.05) in bold.

Normal cognition (NC), Mild cognitive impairment due to Alzheimer’s disease (AD-MCI), Parkinson’s disease with Mild cognitive impairment (PD-MCI), Parkinson’s disease with normal cognition (PD-NC), *p*-values (p), degree of freedom (df), *F*-values (F), Performance of Overall Drawing Score (*VFDB_1_*), Task Performance of Numbers Drawing Score (*VFDB_2_*), Task Performance of Outline Drawing Score (*VFDB_3_*), Task Performance of Clock Hands Drawing Score (*VFDB_4_*), Task Completion Time (*EFDB_1_*), Total Drawing Pause Time (*EFDB_2_*), Initial Drawing Pause Time (*EFDB_3_*), Total Drawing Process Pause Time (*EFDB_4_*), Maximum of Drawing Process Pause Time (*EFDB_5_*), Average of Drawing Process Pause Time (*EFDB_6_*), Number of Pauses during Drawing (*EFDB_7_*), Drawing Time (*EFDB_8_*), Number of Draws (*EFDB_9_*), Efficiency of Drawing (*EFDB_10_*), Length of Drawn Line (*EFDB_11_*), Initial Drawing Speed (*EFDB_12_*), Average of Drawing Speed (*EFDB_13_*), Variability of Drawing Speed (*EFDB_14_*).

In the NC, AD-MCI, PD-MCI and PD-NC groups, there were statistical differences in Task Performance of Overall Drawing Score (*VFDB*_1_) (*p* < 0.001, df = 3), Task Performance of Numbers Drawing Score (*VFDB*_2_) (*p* = 0.002, df = 3), Task Performance of Clock Hands Drawing Score (*VFDB*_4_) (*p* = 0.005, df = 3), Task Completion Time (*EFDB*_1_) (*p* < 0.001, df = 3), Total Drawing Pause Time (*EFDB*_2_) (*p* < 0.001, df = 3), Initial Drawing Pause Time (*EFDB*_3_) (*p* = 0.019, df = 3), Total Drawing Process Pause Time (*EFDB*_4_) (*p* < 0.001, df = 3), Maximum of Drawing Process Pause Time (*EFDB*_5_) (*p* < 0.001, df = 3), Average of Drawing Process Pause Time (*EFDB*_6_) (*p* < 0.001, df = 3), Number of Pauses during Drawing (*EFDB*_7_) (*p* < 0.001, df = 3), Drawing Time (*EFDB*_8_) (*p* < 0.001, df = 3), Number of Draws (*EFDB*_9_) (*p* = 0.029, df = 3), Efficiency of Drawing (*EFDB*_10_) (*p* = 0.002, df = 3, *F* = 5.358), Initial Drawing Speed (*EFDB*_12_) (*p* < 0.001, df = 3, *F* = 8.258), Average of Drawing Speed (*EFDB*_13_) (*p* < 0.001, df = 3) and Variability of Drawing Speed (*EFDB*_14_) (*p* = 0.003, df = 3).

In the NC and AD-MCI groups, Task Performance of Overall Drawing Score (*VFDB*_1_), Task Performance of Numbers Drawing Score (*VFDB*_2_), Task Performance of Clock Hands Drawing Score (*VFDB*_4_), and Efficiency of Drawing (*EFDB*_10_) were significantly lower in the AD-MCI group than in the NC group, whereas the Variability of Drawing Speed (*EFDB*_14_) was significantly higher in the AD-MCI group than in the NC group.

In the NC and PD-MCI groups, Task Completion Time (*EFDB*_1_), Total Drawing Pause Time (*EFDB*_2_), Total Drawing Process Pause Time (*EFDB*_4_), Maximum of Drawing Process Pause Time (*EFDB*_5_), Average of Drawing Process Pause Time (*EFDB*_6_), Number of Pauses during Drawing (*EFDB*_7_), Drawing Time (*EFDB*_8_), and Number of Draws (*EFDB*_9_) were significantly higher in the PD-MCI group than in the NC group. In contrast, Task Performance of Overall Drawing Score (*VFDB*_1_), Task Performance of Numbers Drawing Score (*VFDB*_2_), Task Performance of Clock Hands Drawing Score (*VFDB*_4_), Efficiency of Drawing (*EFDB*_10_), Initial Drawing Speed (*EFDB*_12_), and average speed of drawing in the Average of Drawing Speed (*EFDB*_13_) were significantly lower in the PD-MCI group than in the NC group.

In the NC and PD-NC groups, Task Performance of Overall Drawing Score (*VFDB*_1_) and Initial Drawing Speed (*EFDB*_12_) were significantly lower in the PD-NC group than in the NC group, whereas Number of Pauses during Drawing (*EFDB*_7_) was significantly higher in the PD-NC group than in the NC group.

In the AD-MCI and PD-MCI groups, Task Completion Time (*EFDB*_1_), Total Drawing Pause Time (*EFDB*_2_), Total Drawing Process Pause Time (*EFDB*_4_), Maximum of Drawing Process Pause Time (*EFDB*_5_), Average of Drawing Process Pause Time (*EFDB*_6_), Number of Pauses during Drawing (*EFDB*_7_), and Drawing Time (*EFDB*_8_) were significantly higher in the PD-MCI groups than in the AD-MCI group. In contrast, Initial Drawing Speed (*EFDB*_12_), Average of Drawing Speed (*EFDB*_13_), and Variability of Drawing Speed (*EFDB*_14_) were significantly lower in the PD-MCI group than in the AD-MCI group.

In the PD-MCI and PD-NC groups, Task Completion Time (*EFDB*_1_), Total Drawing Pause Time (*EFDB*_2_), Total Drawing Process Pause Time (*EFDB*_4_), Maximum of Drawing Process Pause Time (*EFDB*_5_), and Average of Drawing Process Pause Time (*EFDB*_6_) were significantly higher in the PD-MCI group than in the PD-NC group. Efficiency of Drawing (*EFDB*_10_), and Average of Drawing Speed (*EFDB*_13_) were significantly lower in the PD-MCI group than in the PD-NC group.

In the AD-MCI and PD-NC groups, Drawing Time (*EFDB*_8_) and Efficiency of Drawing (*EFDB*_10_) were significantly smaller in the AD-MCI group than in the PD-NC group, whereas Initial Drawing Speed (*EFDB*_12_), Average of Drawing Speed (*EFDB*_13_), and Variability of Drawing Speed (*EFDB*_14_) were significantly larger in the AD-MCI group than in the PD-NC group.

### 3.3 Correlation analyses between digital biomarkers and MoCA

We further investigated statistically significant digital biomarkers across the NC, AD-MCI, PD-MCI, and PD-NC groups, specifically focusing on: Performance of Overall Drawing Score (*VFDB*_1_), Task Performance of Numbers Drawing Score (*VFDB*_2_), Task Performance of Clock Hands Drawing Score (*VFDB*_4_), Task Completion Time (*EFDB*_1_), Total Drawing Pause Time (*EFDB*_2_), Initial Drawing Pause Time (*EFDB*_3_), Total Drawing Process Pause Time (*EFDB*_4_), Maximum of Drawing Process Pause Time (*EFDB*_5_), Average of Drawing Process Pause Time (*EFDB*_6_), Number of Pauses during Drawing (*EFDB*_7_), Drawing Time (*EFDB*_8_), Number of Draws (*EFDB*_9_), Efficiency of Drawing (*EFDB*_10_), Initial Drawing Speed (*EFDB*_12_), Average of Drawing Speed (*EFDB*_13_) and Variability of Drawing Speed (*EFDB*_14_). Non-parametric correlations with MoCA total scores and [visuospatial/executive] subtest score were computed using Spearman rank correlation analysis, given non-normal distribution of both variables in all analyzed pairs, as detailed in Table 6.

**Table 6:** Correlation coefficients of digital biomarkers with MoCA total scores and [visuospatial/executive] subtest score.

Digital biomarkers	MoCA score		[Visuospatial/executive] subtest score in MoCA	
	R	*p*	R	*p*
*VFDB*_1_	**0.312**	**<0.001**	**0.472**	**<0.001**
*VFDB*_2_	**0.258**	**0.001**	**0.394**	**<0.001**
*VFDB*_4_	**0.307**	**<0.001**	**0.456**	**<0.001**
*EFDB*_1_	**−0.318**	**<0.001**	**−0.262**	**0.001**
*EFDB*_2_	**−0.343**	**<0.001**	**−0.295**	**<0.001**
*EFDB*_3_	−0.036	0.653	−0.119	0.133
*EFDB*_4_	**−0.384**	**<0.001**	**−0.327**	**<0.001**
*EFDB*_5_	**−0.298**	**<0.001**	**−0.270**	**<0.001**
*EFDB*_6_	**−0.321**	**<0.001**	**−0.231**	**0.003**
*EFDB*_7_	**−0.164**	**0.038**	−0.092	0.247
*EFDB*_8_	−0.072	0.367	−0.066	0.404
*EFDB*_9_	**−0.257**	**0.001**	**−0.265**	**0.001**
*EFDB*_10_	**0.331**	**<0.001**	**0.292**	**<0.001**
*EFDB*_12_	−0.071	0.368	−0.035	0.655
*EFDB*_13_	0.122	0.122	0.090	0.255
*EFDB*_14_	**−0.290**	**<0.001**	**−0.232**	**0.003**

Highlighting significant *p*-values (*p* < 0.05) in bold.

Montreal Cognitive Assessment (MoCA), Spearman rank correlation coefficient (R), *p*-values (*p*), Performance of Overall Drawing Score (*VFDB_1_*), Task Performance of Numbers Drawing Score (*VFDB_2_*), Task Performance of Clock Hands Drawing Score (*VFDB_4_*), Task Completion Time (*EFDB_1_*), Total Drawing Pause Time (*EFDB_2_*), Initial Drawing Pause Time (*EFDB_3_*), Total Drawing Process Pause Time (*EFDB_4_*), Maximum of Drawing Process Pause Time (*EFDB_5_*), Average of Drawing Process Pause Time (*EFDB_6_*), Number of Pauses during Drawing (*EFDB_7_*), Drawing Time (*EFDB_8_*), Number of Draws (*EFDB_9_*), Efficiency of Drawing (*EFDB_10_*), Initial Drawing Speed (*EFDB_12_*), Average of Drawing Speed (*EFDB_13_*), Variability of Drawing Speed (*EFDB_14_*).

Among them, Performance of Overall Drawing Score (*VFDB*_1_) correlated positively with the total MoCA score (Spearman rank correlation coefficient [R] = 0.312, *p* < 0.001) and the [visuospatial/executive] subtest score (*R* = 0.472, *p* < 0.001). Task Performance of Numbers Drawing Score (*VFDB*_2_) correlated positively with the total MoCA score (*R* = 0.258, *p* = 0.001) and the [visuospatial/executive] subtest score (*R* = 0.394, *p* < 0.001). Task Performance of Clock Hands Drawing Score (*VFDB*_4_) correlated positively with the total MoCA score (*R* = 0.307, *p* < 0.001) and the [visuospatial/executive] subtest score (*R* = 0.456, *p* < 0.001). Task Completion Time (*EFDB*_1_) correlated negatively with the total MoCA score (*R* = −0.318, *p* < 0.001) and the [visuospatial/executive] subtest score (*R* = −0.262, *p* = 0.001). Total Drawing Pause Time (*EFDB*_2_) correlated negatively with the total MoCA score (*R* = −0.343, *p* < 0.001) and the [visuospatial/executive] subtest score (*R* = −0.295, *p* < 0.001). Total Drawing Process Pause Time (*EFDB*_4_) correlated negatively with the total MoCA score (*R* = −0.384, *p* < 0.001) and the [visuospatial/executive] subtest score (*R* = −0.327, *p* < 0.001). Maximum of Drawing Process Pause Time (*EFDB*_5_) correlated negatively with the total MoCA score (*R* = −0.298, *p* < 0.001) and the [visuospatial/executive] subtest score (*R* = −0.270, *p* < 0.001). Average of Drawing Process Pause Time (*EFDB*_6_) correlated negatively with the total MoCA score (*R* = −0.321, *p* < 0.001) and the [visuospatial/executive] subtest score (*R* = −0.231, *p* = 0.003). Number of Pauses during Drawing (*EFDB*_7_) correlated negatively with the total MoCA score (*R* = −0.164, *p* = 0.038). Number of Draws (*EFDB*_9_) correlated negatively with the total MoCA score (*R* = −0.257, *p* = 0.001) and the [visuospatial/executive] subtest score (*R* = −0.265, *p* = 0.001). Efficiency of Drawing (*EFDB*_10_) correlated positively with the total MoCA score (*R* = 0.331, *p* < 0.001) and the [visuospatial/executive] subtest score (*R* = 0.292, *p* < 0.001). Variability of Drawing Speed (*EFDB*_14_) correlated negatively with the total MoCA score (*R* = −0.290, *p* < 0.001) and the [visuospatial/executive] subtest score (*R* = −0.232, *p* = 0.003).

### 3.4 Extraction of digital biomarkers of cognitive function and analysis of the ROC curve

We screened 10 digital biomarkers with intergroup variability in AD-MCI and PD-MCI groups. Since the AD-MCI and PD-MCI groups differed in cognitive and motor function, these digital biomarkers may have included both digital biomarkers of cognitive function associated with cognitive impairment and digital biomarkers of motor function associated with motor impairment. Given that the central goal of this study was to investigate the variability in cognitive function between AD-MCI and PD-MCI populations on the clock drawing test, these 10 digital biomarkers were further screened to exclude motor function differences from interfering with cognitive function variability in subsequent analyses.

The 10 digital biomarkers with intergroup variability in the AD-MCI and PD-MCI groups were listed below: Task Completion Time (*EFDB*_1_), Total Drawing Pause Time (*EFDB*_2_), Total Drawing Process Pause Time (*EFDB*_4_), Maximum of Drawing Process Pause Time (*EFDB*_5_), Average of Drawing Process Pause Time (*EFDB*_6_), Number of Pauses during Drawing (*EFDB*_7_), and Drawing Time (*EFDB*_8_), Initial Drawing Speed (*EFDB*_12_), Average of Drawing Speed (*EFDB*_13_) and Variability of Drawing Speed (*EFDB*_14_).

In the PD-MCI and PD-NC groups, there was no statistically significant difference in MDS-UPDRS Part III scores between the two groups, indicating that there were no differences in motor function and only differences in cognitive function between the two groups. Therefore, the 7 digital biomarkers of intergroup variability in PD-MCI and PD-NC groups were digital biomarkers of cognitive function characterizing cognitive function. These included: Task Completion Time (*EFDB*_1_), Total Drawing Pause Time (*EFDB*_2_), Total Drawing Process Pause Time (*EFDB*_4_), Maximum of Drawing Process Pause Time (*EFDB*_5_), and Average of Drawing Process Pause Time (*EFDB*_6_), Efficiency of Drawing (*EFDB*_10_), and Average of Drawing Speed (*EFDB*_13_).

In the PD-NC and NC groups, there were only differences in motor function between the PD-NC and NC groups, the three indicators of intergroup variability in the PD-NC and NC groups were digital biomarkers of motor function characterizing motor function, including Task Performance of Overall Drawing Score (*VFDB*_1_), Number of Pauses during Drawing (*EFDB*_7_) and Initial Drawing Speed (*EFDB*_12_).

**Figure 5:**
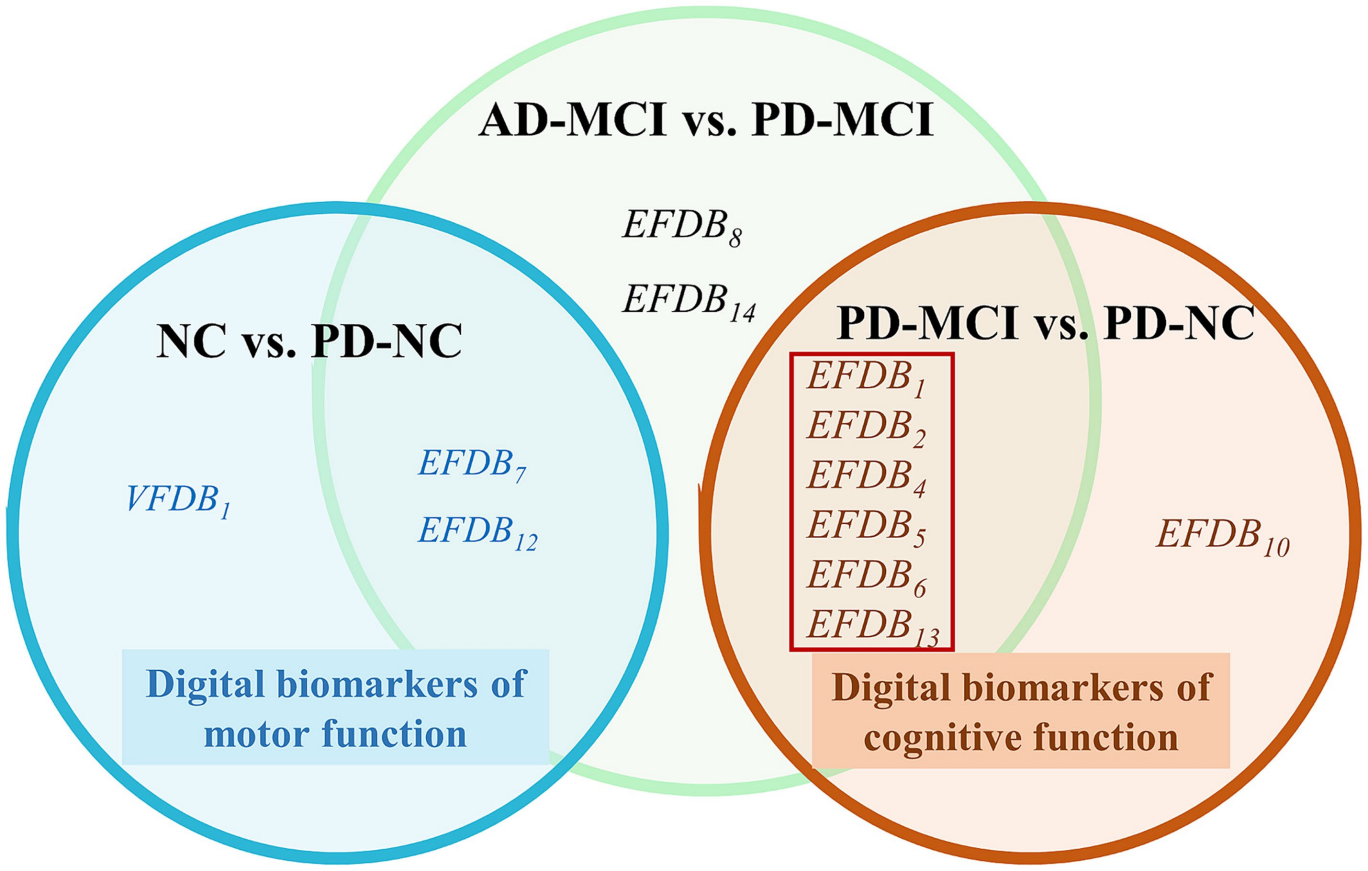
Venn diagram of digital biomarkers. The cognitive functions digital biomarkers in the red boxes that can distinguish between AD-MCI and PD-MCI. Normal cognition (NC), Mild cognitive impairment due to Alzheimer’s disease (AD-MCI), Parkinson’s disease with Mild cognitive impairment (PD-MCI), Parkinson’s disease with normal cognition (PD-NC), Task Performance of Overall Drawing Score (*VFDB*_1_), Task Completion Time (*EFDB*_1_), Total Drawing Pause Time (*EFDB*_2_), Total Drawing Process Pause Time (*EFDB*_4_), Maximum of Drawing Process Pause Time (*EFDB*_5_), Average of Drawing Process Pause Time (*EFDB*_6_), Number of Pauses during Drawing (*EFDB*_7_), and Drawing Time (*EFDB*_8_), Efficiency of Drawing (*EFDB*_10_), Initial Drawing Speed (*EFDB*_12_), Average of Drawing Speed (*EFDB*_13_), Variability of Drawing Speed (*EFDB*_14_).

To identify digital biomarkers that could characterize cognitive function in the AD-MCI and PD-MCI groups, we plotted Venn diagrams, the Venn diagram of digital biomarkers is shown in Figure 5.

In Figure 5, we found no overlap in digital biomarkers of cognitive function between PD-MCI and PD-NC groups, or in digital biomarkers of motor function between NC and PD-NC groups. This indicates that biomarkers identified in the PD-MCI and PD-NC groups accurately reflect cognitive function, while those in the NC and PD-NC groups accurately reflect motor function. Finally, we identified six digital biomarkers of cognitive function that could accurately characterize AD-MCI and PD-MCI populations as follows:

Task Completion Time (*EFDB*_1_), Total Drawing Pause Time (*EFDB*_2_), Total Drawing Process Pause Time (*EFDB*_4_), Maximum of Drawing Process Pause Time (*EFDB*_5_), Average of Drawing Process Pause Time (*EFDB*_6_), and Average of Drawing Speed (*EFDB*_13_).

**Figure 6:**
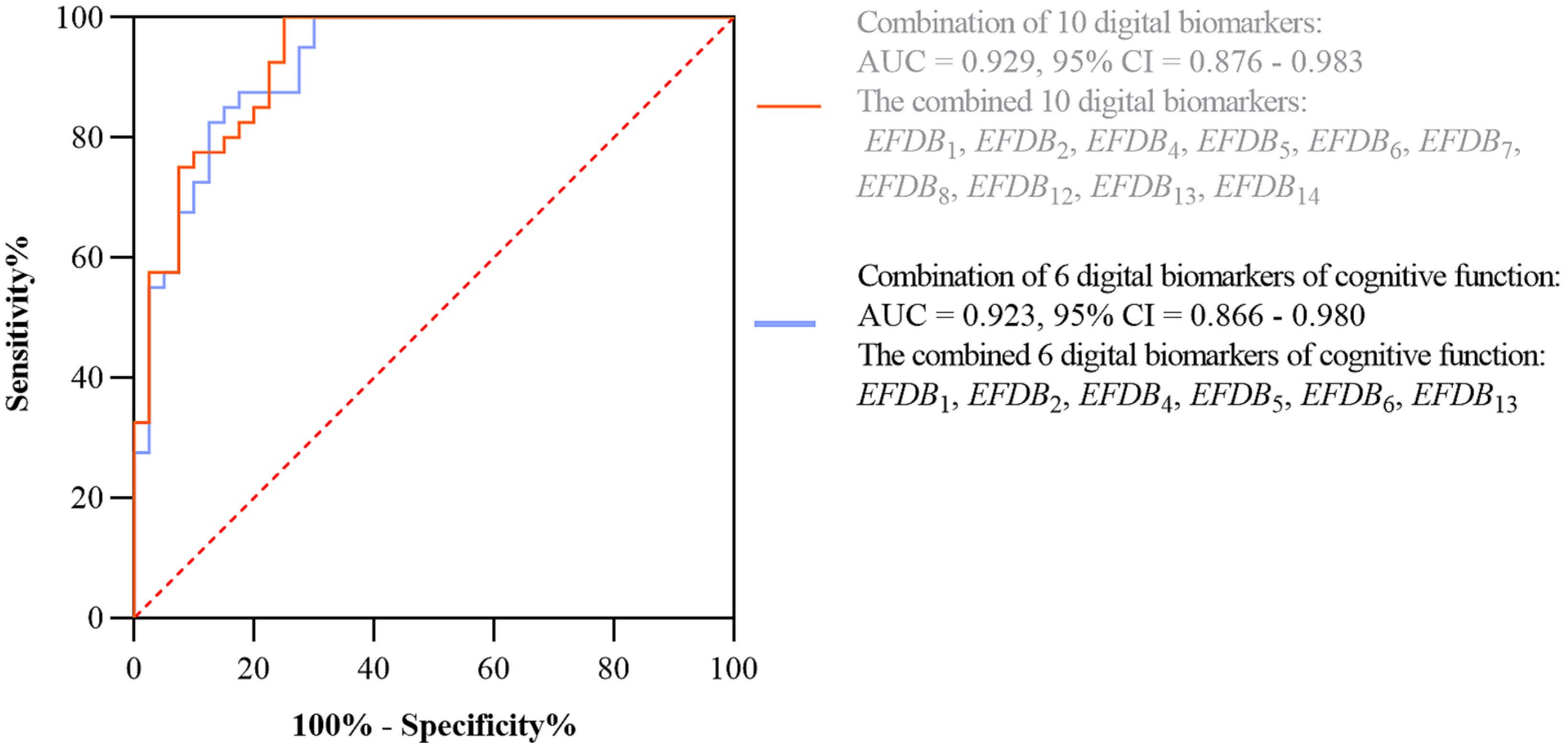
ROC curves and 95% CI of combined digital biomarkers distinguishing AD-MCI and PD-MCI groups. Area under the roc curve (AUC), confidence interval (CI), Task Completion Time (*EFDB*_1_), Total Drawing Pause Time (*EFDB*_2_), Total Drawing Process Pause Time (*EFDB*_4_), Maximum of Drawing Process Pause Time (*EFDB*_5_), Average of Drawing Process Pause Time (*EFDB*_6_), Number of Pauses during Drawing (*EFDB*_7_), and Drawing Time (*EFDB*_8_), Initial Drawing Speed (*EFDB*_12_), Average of Drawing Speed (*EFDB*_13_), Variability of Drawing Speed (*EFDB*_14_).

Subsequently, we plotted ROC curves to assess the ability of digital biomarkers to differentiate the AD-MCI group from the PD-MCI group. The combined AUC of the six digital biomarkers of cognitive function was 0.923, 95% confidence interval (CI) = 0.876–0.983, which was only slightly lower than the combined AUC of the 10 digital biomarkers with intergroup variability (AUC = 0.929, 95% CI: 0.866–0.908). The ROC curves and 95% CI of the combined digital biomarkers that differentiate the AD-MCI and PD-MCI groups are shown in Figure 6.

### 3.5 Differential analysis and ROC analysis of digital biomarkers of cognitive function in highly educated individuals in the AD-MCI and PD-MCI groups

Considering that there was an effect of literacy on cognitive functioning, we screened highly educated individuals (years of education ≥12 years) in the AD-MCI and PD-MCI groups and divided them into AD-MCI_1_ and PD-MCI_1_ groups for differential analyses of demographic and numerical biomarkers. Among them, age (*p* = 0.348, *t* = 0.947), sex (*p* = 0.535), years of education (*p* = 0.368), and MMSE (*p* = 0.500) were not statistically different. MoCA (*p* = 0.047) was statistically different. Digital biomarkers included only the above obtained digital biomarkers of cognitive function. In particular, Task Completion Time (*EFDB*_1_) (*p* < 0.001), Total Drawing Pause Time (*EFDB*_2_) (*p* < 0.001), Total Drawing Process Pause Time (*EFDB*_4_) (*p* = 0.001), Maximum of Drawing Process Pause Time (*EFDB*_5_) (*p* = 0.026), Average of Drawing Process Pause Time (*EFDB*_6_) (*p* = 0.001), and Average of Drawing Speed (*EFDB*_13_) (*p* < 0.001, *t* = 6.038) were statistically different. The results of the differential analysis of demography and digital biomarkers of cognitive function are shown in Tables 7, 8.

**Table 7:** Results of demographic difference analysis between the AD-MCI_1_ and PD-MCI_1_ groups.

	AD-MCI_1_ (*n* = 28)	PD-MCI_1_ (*n* = 31)	*p* value, t*
Age, years	66.39 ± 6.68	64.58 ± 7.88	0.348, 0.947
Sex (female/male)	14/14	18/13	0.535
Years of education	12.00 (3.00)	12.00 (3.00)	0.368
MMSE	26.00 (2.00)	26.00 (3.00)	0.500
MoCA	22.00 (3.75)	21.00 (5.00)	0.047

* means when variables satisfy a normal distribution using an independent samples t-test, *p*-values and *t*-values will be presented.

Mild cognitive impairment due to Alzheimer’s disease (AD-MCI), Parkinson’s disease with Mild cognitive impairment (PD-MCI), participants with years of education ≥ 12 years in the AD-MCI group (AD-MCI_1_), participants with years of education ≥ 12 years in the AD-MCI group (PD-MCI_1_), *p*-values (p), *t*-values (t), Minimum Mental State Examination (MMSE), Montreal Cognitive Assessment (MoCA).

**Table 8:** Results of the differential analysis of digital biomarkers of cognitive function between the AD-MCI_1_ and PD-MCI_1_ groups.

Digital biomarkers of cognitive function	AD-MCI_1_ (*n* = 28)	PD-MCI_1_ (*n* = 31)	*p* value, t*
*EFDB*_1_	42.89 (28.84)	73.14 (40.38)	**<0.001**
*EFDB*_2_	25.15 (23.35)	48.07 (37.34)	**<0.001**
*EFDB*_4_	21.19 (21.91)	42.45 (35.31)	**0.001**
*EFDB*_5_	4.78 (10.58)	10.34 (13.12)	**0.026**
*EFDB*_6_	0.85 (0.53)	1.40 (1.17)	**0.001**
*EFDB*_13_	321.55 ± 115.14	180.21 ± 48.05	**<0.001**, 6.038

* means when variables satisfy a normal distribution using an independent samples t-test, *p*-values and *t*-values will be presented. Highlighting significant *p*-values (*p* < 0.05) in bold.

Mild cognitive impairment due to Alzheimer’s disease (AD-MCI), Parkinson’s disease with Mild cognitive impairment (PD-MCI), participants with years of education ≥ 12 years in the AD-MCI group (AD-MCI_1_), participants with years of education ≥ 12 years in the AD-MCI group (PD-MCI_1_), *p*-values (p), *t*-values (t), Task Completion Time (*EFDB_1_*), Total Drawing Pause Time (*EFDB_2_*), Total Drawing Process Pause Time (*EFDB_4_*), Maximum of Drawing Process Pause Time (*EFDB_5_*), Average of Drawing Process Pause Time (*EFDB_6_*), Average of Drawing Speed (*EFDB_13_*).

**Figure 7:**
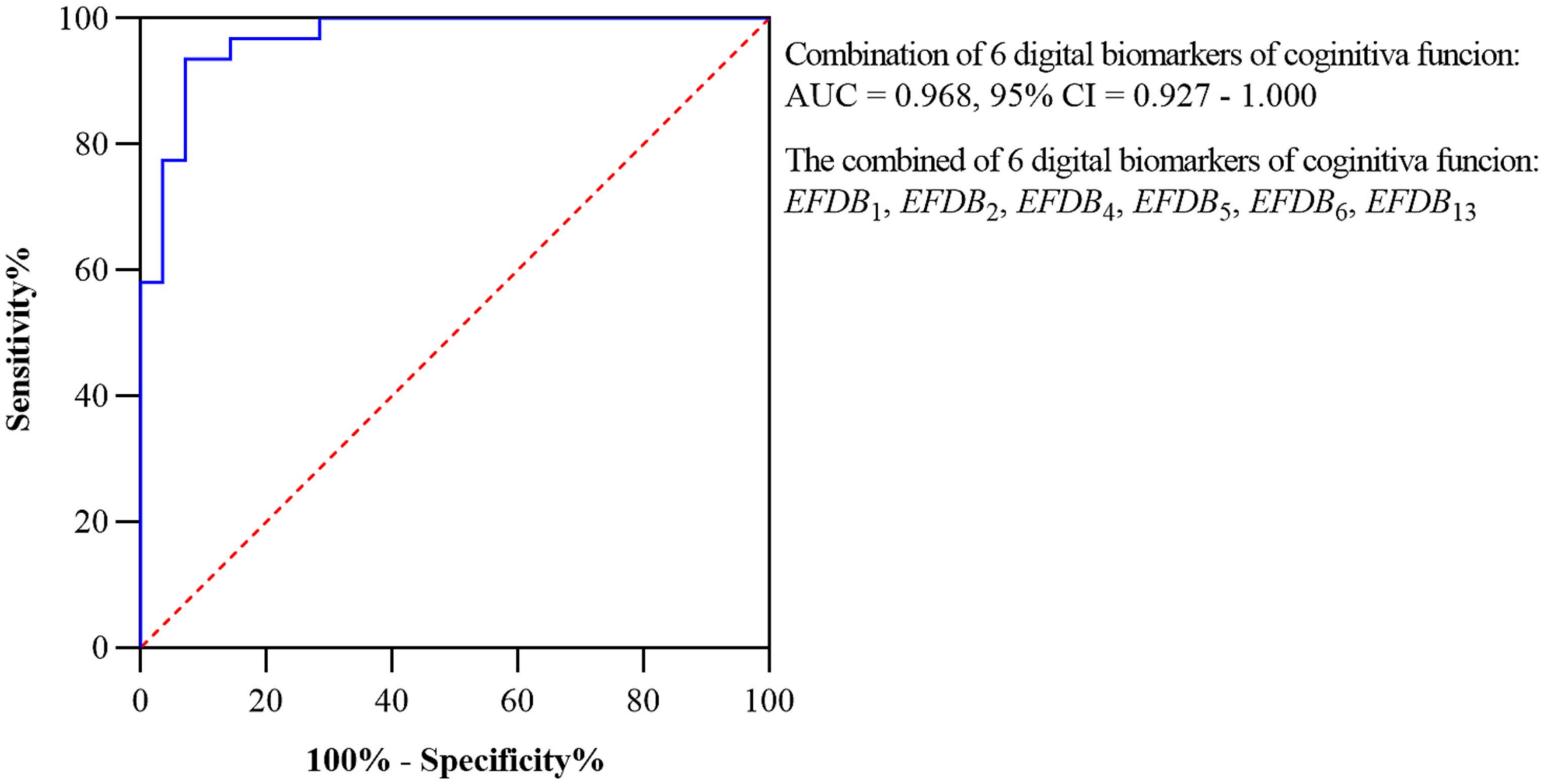
ROC curves and 95% CI of combined digital biomarkers distinguishing AD-MCI_1_ and PD-MCI_1_ groups. Area under the roc curve (AUC), confidence interval (CI), Task Completion Time (*EFDB*_1_), Total Drawing Pause Time (*EFDB*_2_), Total Drawing Process Pause Time (*EFDB*_4_), Maximum of Drawing Process Pause Time (*EFDB*_5_), Average of Drawing Process Pause Time (*EFDB*_6_), Average of Drawing Speed (*EFDB*_13_).

Subsequently, we plotted ROC curves to assess the ability of the digital biomarkers to distinguish the AD-MCI_1_ group from the PD-MCI_1_ group. The joint AUC of the above six statistically preexisting digital biomarkers of cognitive function was 0.968, CI = 0.927–1.000. The ROC curves and 95% CI for the combined digital biomarkers to differentiate the AD-MCI_1_ group from the PD-MCI_1_ group are shown in Figure 7.

## 4 Discussion

In this study, we proposed the research hypothesis that AD-MCI and PD-MCI populations exhibit different cognitive functioning characteristics on the dCDT, and that the two populations can be effectively distinguished based on this characteristic. Based on this hypothesis, we designed the dCDT to characterize and quantify differences in cognitive functioning between AD-MCI and PD-MCI populations at a fine-grained level. We extracted statistically different digital biomarkers between AD-MCI, PD-MCI, PD-NC and NC groups, respectively. The digital biomarkers were differentiated in terms of motor and cognitive functions, and ultimately six digital biomarkers of cognitive functions were screened from the AD-MCI and PD-MCI groups. The combined AUC of six digital biomarkers for distinguishing between AD-MCI and PD-MCI groups could reach 0.923.

As a multistep cognitive function assessment tool that integrates certain sequences, the clock drawing test necessitates the synergy of multiple cognitive and motor functions like executive functions and visuospatial abilities (Müller et al., 2017). Specifically, during the clock-drawing process, as the participants touches the screen to draw the clock, their executive functions like executive control and cognitive dexterity become dominant. This dynamic process can be accurately characterized by digital biomarkers like drawing duration, speed, and efficiency (Dion et al., 2020). In addition, the clock-drawing test demands that participants to draw circular or near-circular outline and correctly place the numerals as well as the clock hands. These details assess participants’ visuospatial cognitive abilities, characterized by digital biomarkers like the outline scores, numbers scores, and hand-drawing scores (Davoudi et al., 2020). Thus, by synthesizing and analyzing digital biomarkers from the dCDT, participants’ cognitive and motor performance in complex tasks can be finely characterized.

First, the results of this study showed that Initial Drawing Speed (*EFDB*_12_), Average of Drawing Speed (*EFDB*_13_), Variability of Drawing Speed (*EFDB*_14_) were significantly lower in the PD-MCI group than the AD-MCI group. These results were consistent with those of Saur (Saur et al., 2012). Previous studies have shown that PD-MCI patients have more severe impairments in executive functioning than AD-MCI patients. Moreover, executive dysfunction, which is the most characteristic cognitive impairment in PD-MCI patients, is closely related to impaired integrity of the frontal-striatal loop (van den Heuvel et al., 2013). In contrast to the pattern of cognitive impairment in PD-MCI patients, the pattern of cognitive decline in AD-MCI patients was primarily associated with cortical involvement in the hippocampus and medial temporal lobe (Li et al., 2022). Executive dysfunction significantly affects patients’ social behavior, making it the most common clinical complaint. This was usually manifested as a greater difficulty in completing daily and routine tasks. Additionally, the impairment of executive functioning was particularly prominent when performing complex tasks that required the integration of multiple sequential steps (Blair, 2016). Therefore, in the dCDT, the executive functions of PD-MCI patients may be more severely impaired than those of AD-MCI patients, resulting in significantly slower Average of Drawing Speed (*EFDB*_13_).

In addition, our findings showed that Task Completion Time (*EFDB*_1_), Total Drawing Pause Time (*EFDB*_2_), Total Drawing Process Pause Time (*EFDB*_4_), Maximum of Drawing Process Pause Time (*EFDB*_5_), Average of Drawing Process Pause Time (*EFDB*_6_), Number of Pauses during Drawing (*EFDB*_7_), and Drawing Time (*EFDB*_8_) were significantly higher in the PD-MCI groups than in the AD-MCI group. This suggested that PD-MCI patients have more significant deficits in executive ability than AD-MCI patients.

Whereas the PD-MCI and AD-MCI groups showed differential digital biomarkers on the clock drawing test, which may have resulted from cognitive differences or motor differences between them. The aim was to further explore and identify digital biomarkers to accurately characterize cognitive functioning differences in the clock drawing test between AD-MCI and PD-MCI populations. We separately compared cognitive functioning differences between the PD-MCI and PD-NC groups (no statistically significant difference in MDS-UPDRS-III motor scores between the two groups) and between PD-NC and NC groups (no statistically significant difference in MMSE and MoCA scores between the two groups). This was done with the objective to identifying digital biomarkers of cognitive function that could accurately differentiate between the PD-MCI and AD-MCI group.

In the PD-MCI and PD-NC groups, there were only differences in cognitive function between the two groups, so digital biomarkers that were significantly different between the two groups can be considered digital biomarkers of cognitive function characterizing cognitive function differences. Among the digital biomarkers, Task Completion Time (*EFDB*_1_), Total Drawing Pause Time (*EFDB*_2_), Total Drawing Process Pause Time (*EFDB*_4_), Maximum of Drawing Process Pause Time (*EFDB*_5_), and Average of Drawing Process Pause Time (*EFDB*_6_) were significantly higher in the PD-MCI group than in the PD-NC group. Efficiency of Drawing (*EFDB*_10_), and Average of Drawing Speed (*EFDB*_13_) were significantly lower in PD-MCI group than in PD-NC group. These findings were largely consistent with Cosgrove et al. and together revealed significant deficits in executive function in PD-MCI patients (Cosgrove et al., 2021). These results further validated that digital biomarkers like pause duration and drawing speed effectively capture executive function deficits.

In the PD-NC and NC groups, digital biomarkers that were significantly different between the two groups can be considered digital biomarkers of motor function that characterize motor function, since there were only differences in motor function between the two groups. The results of the study showed that the PD-NC group had a significantly higher Number of Pauses during Drawing (*EFDB*_7_) and slower Initial Drawing Speed (*EFDB*_12_) compared to the NC group. This difference may be related to motor retardation and reduced motor control in PD-NC populations.

Based on the above findings of the PD-MCI and AD-MCI groups, the PD-MCI and PD-NC groups, and the PD-NC and NC groups, as well as the cascading relationships between the three groups of digital biomarkers, we finally identified six digital biomarkers of cognitive function that were able to accurately reflect the differences in cognitive function between AD-MCI and PD-MCI populations. These 6 digital biomarkers of cognitive function include: Task Completion Time (*EFDB*_1_), Total Drawing Pause Time (*EFDB*_2_), Total Drawing Process Pause Time (*EFDB*_4_), Maximum of Drawing Process Pause Time (*EFDB*_5_), Average of Drawing Process Pause Time (*EFDB*_6_), and Average of Drawing Speed (*EFDB*_13_). The combined efficacy of these six digital biomarkers of cognitive function in distinguishing between AD-MCI patients and PD-MCI patients was up to 0.923, which further confirms that there are indeed differences in cognitive function between the AD-MCI populations and the PD-MCI populations in the clock-drawing test, and that by using these digital biomarkers of cognitive function, we can more accurately differentiate between the two populations. At the same time, considering the influence of literacy on cognitive function, we performed differential and ROC analyses of digital biomarkers of cognitive function in highly educated individuals in both AD-MCI and PD-MCI groups (given that the total sample size of the low-education group was too small, no analysis was performed). Six statistically significant digital biomarkers of cognitive function were finally screened, and their joint warning AUC was 0.968, which was higher than the original joint warning AUC = 0.923, suggesting that the results of this study were to some extent influenced by literacy. Therefore, future studies still need to include more groups with different literacy levels to clarify the extent to which literacy influences this study.

In addition, the differential results of the NC and AD-MCI groups, the NC and PD-MCI groups, and the AD-MCI and PD-NC groups in the dCDT were investigated. In comparisons between the AD-MCI and NC groups, we found that Task Performance of Overall Drawing Score (*VFDB*_1_), Task Performance of Numbers Drawing Score (*VFDB*_2_), Task Performance of Clock Hands Drawing Score (*VFDB*_4_), and Efficiency of Drawing (*EFDB*_10_) were significantly lower in the AD-MCI group than in the NC group, whereas the Variability of Drawing Speed (*EFDB*_14_) was significantly higher in the AD-MCI group than in the NC group. Significant deficits in executive function and visuospatial abilities were confirmed in AD-MCI patients.

In the NC and the PD-MCI groups, we found that Task Completion Time (*EFDB*_1_), Total Drawing Pause Time (*EFDB*_2_), Total Drawing Process Pause Time (*EFDB*_4_), Maximum of Drawing Process Pause Time (*EFDB*_5_), Average of Drawing Process Pause Time (*EFDB*_6_), Number of Pauses during Drawing (*EFDB*_7_), Drawing Time (*EFDB*_8_), and Number of Draws (*EFDB*_9_) were significantly higher in the PD-MCI group than in the NC group. In contrast, Task Performance of Overall Drawing Score (*VFDB*_1_), Task Performance of Numbers Drawing Score (*VFDB*_2_), Task Performance of Clock Hands Drawing Score (*VFDB*_4_), Efficiency of Drawing (*EFDB*_10_), Initial Drawing Speed (*EFDB*_12_), and average speed of drawing in the Average of Drawing Speed (*EFDB*_13_) were significantly lower in the PD-MCI group than in the NC group. These findings confirmed the significant deficits in visuospatial and executive functions in patients with PD-MCI.

In the AD-MCI and the PD-NC groups, we found that Initial Drawing Speed (*EFDB*_12_), Average of Drawing Speed (*EFDB*_13_), and Variability of Drawing Speed (*EFDB*_14_) were significantly larger in the AD-MCI group than in the PD-NC group. This showed that AD-MCI group were significantly faster than the PD-NC group, suggesting cognitive decline in AD-MCI and decreased fine motor control in PD-NC. Variability of Drawing Speed (*EFDB*_14_) illustrated the stability of participants’ drawing speed, and we suggest that AD-MCI may be less stable than PD-MCI under cognitive task (Montero-Odasso et al., 2014).

To further demonstrate that the above digital biomarkers characterize cognitive functions well, we performed a correlation analysis between the digital biomarkers and the MoCA scale. Performance of Overall Drawing Score (*VFDB*_1_), Task Performance of Numbers Drawing Score (*VFDB*_2_), Task Performance of Clock Hands Drawing Score (*VFDB*_4_) and Efficiency of Drawing (*EFDB*_10_) were positively correlated with the MoCA scale as well as the [visuospatial/executive] subtest score; Task Completion Time (*EFDB*_1_), Total Drawing Pause Time (*EFDB*_2_), Total Drawing Process Pause Time (*EFDB*_4_), Maximum of Drawing Process Pause Time (*EFDB*_5_), Number of Pauses during Drawing (*EFDB*_7_), Number of Draws (*EFDB*_9_) and Variability of Drawing Speed (*EFDB*_14_) were negatively correlated with the MoCA scale as well as with the [visuospatial/executive] subtest score. The results suggested that the above mentioned digital biomarkers can better characterize visuospatial and executive functions, providing a new tool for early screening and dynamic monitoring of cognitive impairment.

The effects of Parkinson’s disease (PD) drugs on motor function and cognitive processing speed are complex. It was suggested that decreased processing speed in PD patients is associated with abnormalities in the caudate nucleus, and that drugs may affect these regions (Price et al., 2016). There were also studies that mentioned the effects of dopamine medications on executive function and error processing, such as abnormal ERN waves, which might affect cognitive control (Seer et al., 2017; Yang et al., 2017). However, some studies have also found that processing speed is associated with reduced FDOPA uptake in the caudate nucleus, and medication may not fully restore this function (Pal et al., 2016). Most of the patients included in this study used medication, but were in OFF medication at the time of testing, which reduced the drug’s effect to some extent.

Compared to previous studies, the main innovation of this study is that digital biomarkers that can accurately characterize participants’ cognitive functions were extracted through the dCDT, thus providing a fine-grained quantification of participants’ cognitive functioning characteristics during task execution. Moreover, this dCDT demonstrated a good discriminatory ability to distinguish between AD-MCI and PD-MCI populations. Although previous studies also used digital clock drawing tests to differentiate AD patients from PD patients/PD-MCI patients, these studies had significant limitations, such as a single dimension of extracted metrics, low discriminatory efficacy, and a limited sample size (Allone et al., 2018). In contrast, the dCDT proposed in this study not only had higher accuracy, but also significantly improved assessment efficiency, which could be completed in just 3 min, much less than the time-consuming traditional scale assessment methods. Therefore, the dCDT proposed in this study was high objectivity, accuracy and efficiency, with potential for in-depth research and wide dissemination, and was relatively unaffected by race, culture and language compared to neuropsychological scales such as MMSE, MoCA, and others (Kehl-Floberg et al., 2023). In addition, compared to our team’s previous study (which demonstrated whether there was a difference in cognitive function between NC and AD-MCI through digital biomarkers), this study built on pre-existing algorithms to differentiate between different types of cognitive dysfunction (AD-MCI and PD-MCI) through digital biomarkers. Therefore, the dCDT proposed in this study, which was highly objective, less time-consuming, had good replication potential in densely populated communities, and provided a new approach to differentiate between AD-MCI and PD-MCI using digital biomarkers in initial community screening.

However, this study also presents some limitations. First, the effective sample size included in this study was 161 cases, and all were from a single medical center, potentially limiting the generalizability of the findings to the overall AD-MCI and PD-MCI populations. To overcome this limitation, future research aims to conduct a multicenter study and increase the sample size, enhancing the accuracy and generalizability of the study results. Second, potential confounders such as gender and cognitive drugs (e.g., dopaminergic treatments in PD patients), which are limited by the design of the study and scope of data collection, have not been systematically addressed and may have biased trial results. Third, the dimensions of the digital biomarkers explored in this study are limited, and future research will explore additional dimensions such as pressure, orientation, acceleration, angular velocity, and delving into the medical mechanisms and potential associations with blood biomarkers or imaging biomarkers. Meanwhile, the dCDT designed in this study is primarily suited for early warning and screening of cognitive disorders, but for diagnostic use in AD-MCI and PD-MCI, further integration with multidimensional data (such as Aβ-PET, MMSE, MoCA) is required. However, it is hoped that the diagnosis of AD-MCI and PD-MCI may be achieved by dCDT alone at a later stage as more data are recorded and combined with large model technology. Finally, this study is currently limited to a cross-sectional study due to research conditions. In order to verify the validity and reliability of the dCDT more comprehensively, future research will involve longitudinal studies.

## 5 Conclusion

In summary, we proposed the research hypothesis that AD-MCI and PD-MCI populations exhibit different cognitive functioning characteristics in the digital clock drawing test, and that based on this characteristic, we can effectively differentiate between these two populations. Based on this hypothesis, we designed the dCDT, extracted digital biomarkers that can characterize participants’ cognitive functions, and quantified participants’ task-wide cognitive function characteristics at a fine-grained level, revealing differences in cognitive functions between AD-MCI populations and PD-MCI populations. After clinical validation, the AUC of digital biomarkers of cognitive function in distinguishing between AD-MCI and PD-MCI populations was up to 0.923, and the method provided a favorable reference for early diagnosis, treatment and prevention of dementia development in AD-MCI and PD-MCI populations.

## Data Availability

The raw data supporting the conclusions of this article will be made available by the authors, without undue reservation.

## Supplementary material

SUPPLEMENTARY FIGURE 1 Participant screening process. Normal cognition (NC), Mild cognitive impairment due to Alzheimer’s disease (AD-MCI), Parkinson’s disease with Mild cognitive impairment (PD-MCI), Parkinson’s disease with normal cognition (PD-NC).

SUPPLEMENTARY FIGURE 2 The calculation of Task Performance Calculation of Clock Hands Drawing Score(VFDB4). Φ was the classification network.
