# Supplementary figures and images for: Differential cognitive functioning in the digital clock drawing test in AD-MCI and PD-MCI populations
Source: Front Neurosci. 2025 Mar 13;19:1558448. doi: 10.3389/fnins.2025.1558448 (PMC11965901; doi:10.3389/fnins.2025.1558448)

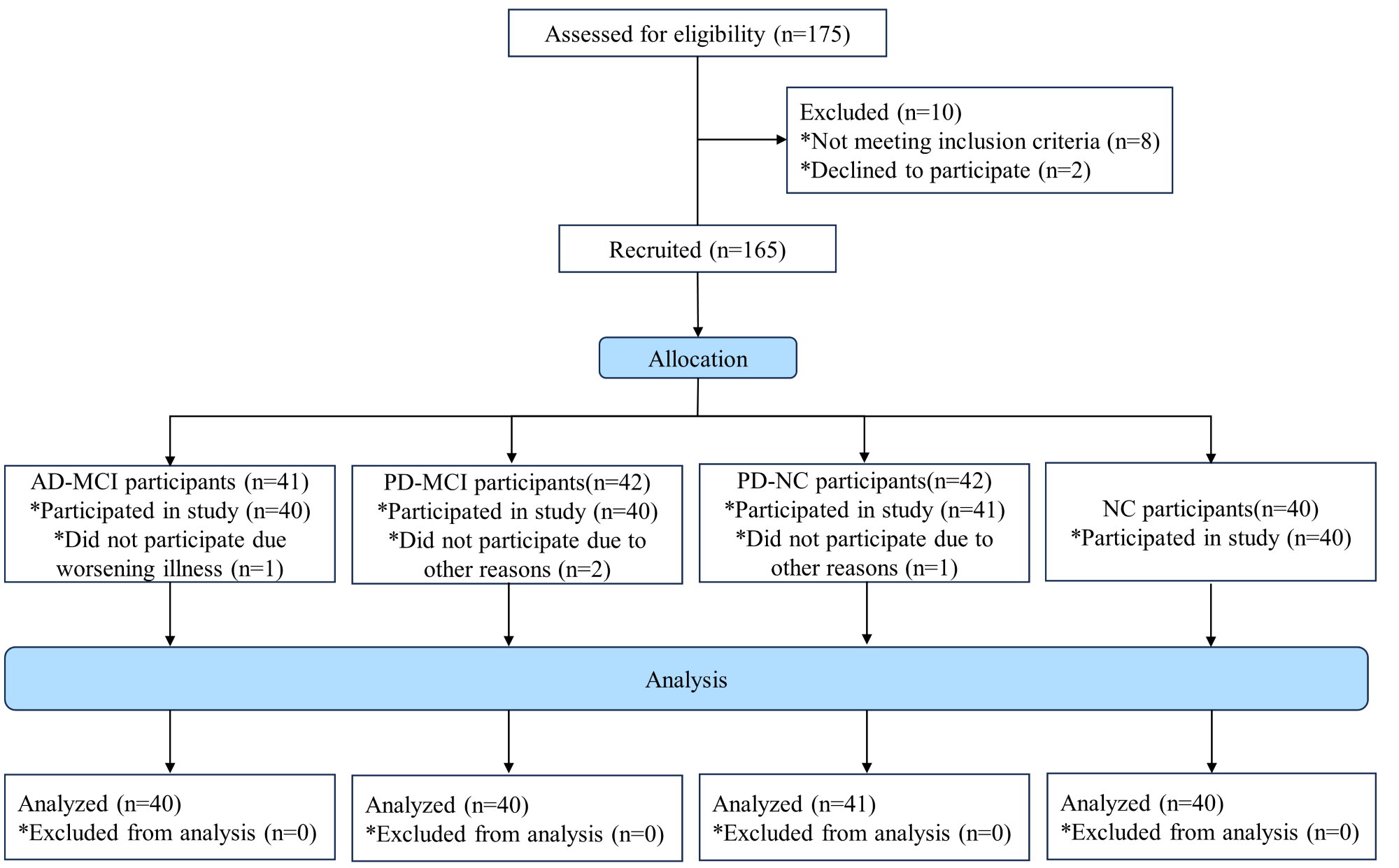

Supplement: SUPPLEMENTARY FIGURE 1 — Participant screening process. Normal cognition (NC), Mild cognitive impairment due to Alzheimer’s disease (AD-MCI), Parkinson’s disease with Mild cognitive impairment (PD-MCI), Parkinson’s disease with normal cognition (PD-NC). [file Image_1.tif]

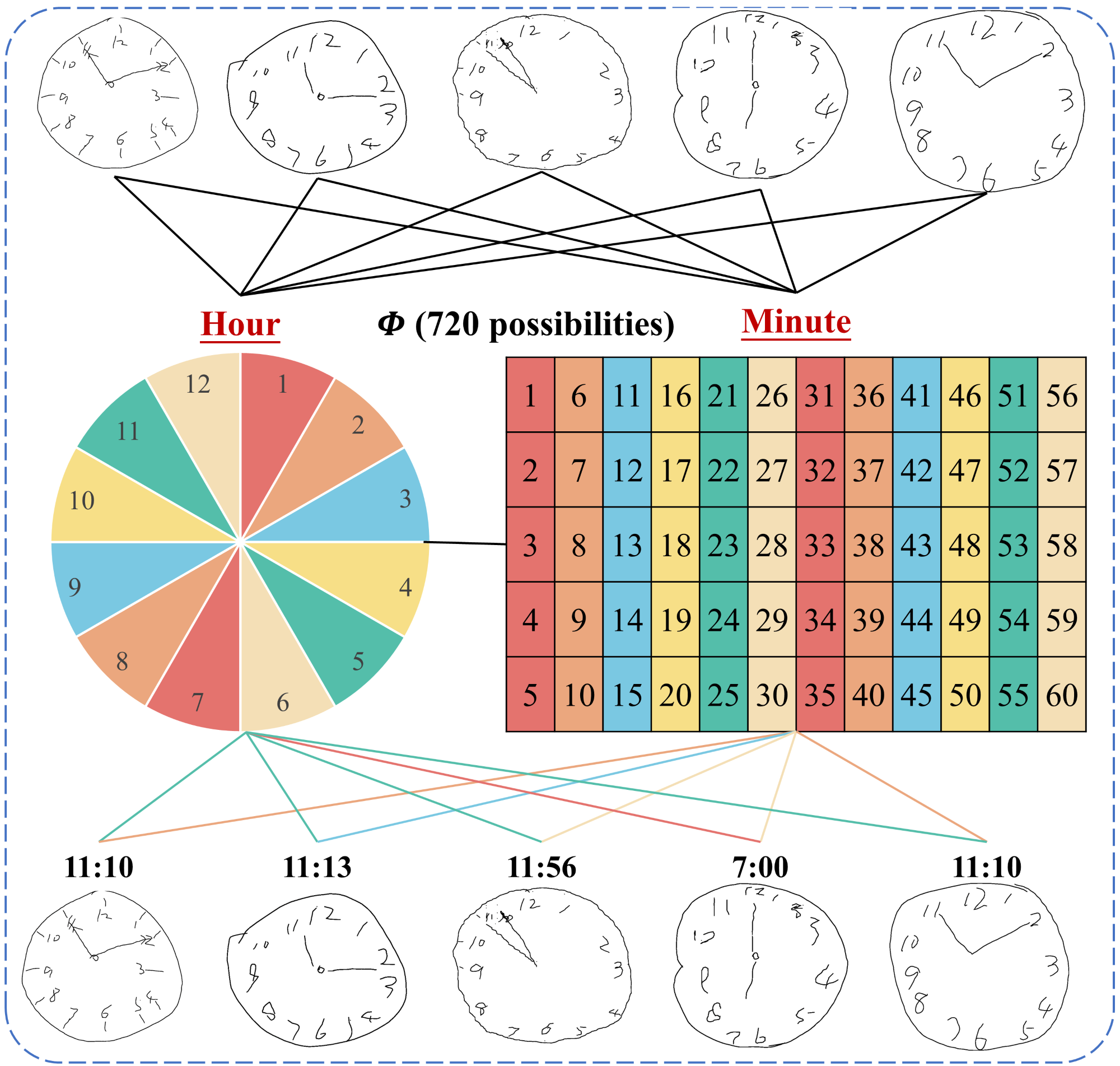

Supplement: SUPPLEMENTARY FIGURE 2 — The calculation of Task Performance Calculation of Clock Hands Drawing Score(VFDB4). Φ was the classification network. [file Image_2.tif]
