# Supplementary material for: Differential cognitive functioning in the digital clock drawing test in AD-MCI and PD-MCI populations
Source: Front Neurosci. 2025 Mar 13;19:1558448. doi: 10.3389/fnins.2025.1558448 (PMC11965901; doi:10.3389/fnins.2025.1558448)
Supplement: Supplementary file 3 [file Table_1.docx]

Supplementary Material

# Supplementary Figures and Tables

## Supplementary Tables

Supplementary Table 1. The demographic characteristics of the participants

|  | **NC**  **(n =** 40**)** | **AD-MCI**  **(n =** 40**)** | **PD-MCI**  **(n =** 40**)** | **PD-NC**  **(n =** 41**)** |
| --- | --- | --- | --- | --- |
| Age, years | 61.00(15.25) | 65.50(13.00) | 66.00(12.00) | 65.00(9.00) |
| Sex (female/male) | 22/18 | 21/19 | 21/19 | 17/24 |
| Years of education | 12.00(6.00) | 12.00(6.00) | 12.00(0.00) | 12.00(3.50) |
| MMSE | 29.00(2.00) | 25.50(2.00) | 26.00(2.75) | 28.00(1.50) |
| MoCA | 25.00(3.00) | 22.00(3.00) | 21.00(4.75) | 25.00(1.00) |
| MDS-UPDRS Ⅲ | 4.00(2.00) | / | 19.50(12.75) | 16.00(8.00) |
| Course of disease | / | 2.00(0.50) | 2.55(0.63) | 2.50(0.50) |

Normal cognition (NC), Mild cognitive impairment due to Alzheimer's disease (AD-MCI), Parkinson's disease with Mild cognitive impairment (PD-MCI), Parkinson's disease with normal cognition (PD-NC), *p*-values (*p*), degree of freedom(df), Minimum Mental State Examination (MMSE), Montreal Cognitive Assessment (MoCA), Movement Disorder Society Unified Parkinson's Disease Rating Scale III (MDS-UPDRS III).
